# Differential Effects of Ethical Education, Physical Hatha Yoga, and Mantra Meditation on Well-Being and Stress in Healthy Participants—An Experimental Single-Case Study

**DOI:** 10.3389/fpsyg.2021.672301

**Published:** 2021-08-05

**Authors:** Karin Matko, Peter Sedlmeier, Holger C. Bringmann

**Affiliations:** ^1^Department of Psychology, Chemnitz University of Technology, Chemnitz, Germany; ^2^Institute for Social Medicine, Epidemiology and Health Economics, Charité-Universitätsmedizin, Corporate Member of Freie Universität Berlin, Humboldt-Universität zu Berlin and Berlin Institute of Health, Berlin, Germany; ^3^Department of Psychiatry, Psychosomatics and Psychotherapy, Diakoniekliniken Zschadrass, Colditz, Germany

**Keywords:** yoga components, mantra meditation, ethical education, differential effects, incremental effects, single-case research, multilevel modeling, mixed-method

## Abstract

Traditionally, yoga is a multicomponent practice consisting of postures, breathing techniques, meditation, mantras, and ethics. To date, only a few studies have tried to dismantle the effects of each of these components and their combinations. To fill this gap, we examined the incremental effects of ethical education and physical Hatha yoga on mantra meditation using a single-case multiple-baseline design. This study was part of a project evaluating the new mind–body program *Meditation-Based Lifestyle Modification*. Fifty-seven healthy participants with no regular yoga or meditation practice were randomly assigned to three baselines (7, 14, and 21 days) and four conditions using a random number generator. The conditions were mantra meditation alone (MA), meditation plus physical yoga (MY), meditation plus ethical education (ME), and meditation plus yoga and ethical education (MYE). All the interventions lasted for 8 weeks and were run consecutively according to baseline length. During the baseline and treatment phases, participants received daily questionnaires measuring their well-being (WHO-5 Well-Being Index), stress (Perceived Stress Scale), and subjective experiences. Forty-two participants completed the treatment and were entered in the analyses. We analyzed our data using visual inspection, effect size estimation (Tau-*U*), and multilevel modeling. Almost all participants showed a longitudinal increase in well-being. Regarding between-group differences, participants who received ethical education exhibited the largest increases in well-being (Tau-*U* = 0.30/0.23 for ME/MYE), followed by participants in the MY condition (Tau-*U* = 0.12). Conversely, participants in the MA condition showed no change (Tau-*U* = 0.07). There was a tendency for the combined treatments to decrease stress. This tendency was strongest in the MY condition (Tau-*U* = –0.40) and reversed in the MA condition (Tau-*U* = 0.17). These results emphasize the incremental and differential effects of practicing meditation in combination with other practices from the eight-fold yoga path. This approach is valuable for better understanding the multifaceted practice of yoga.

**Clinical Trial Registration:**www.ClinicalTrials.gov, identifier: NCT04252976.

## Introduction

Yoga originates from a rich and ancient spiritual tradition that encompasses a variety of diverse practices, such as physical postures, breathing techniques, meditation techniques, mantras, and ethical teachings (Feuerstein, 2012; Telles and Singh, 2013). These practices are designed to promote personal and spiritual growth with the ultimate aim of gaining access to pure consciousness and reaching “enlightenment” (Sedlmeier and Srinivas, 2019). A growing number of studies are acknowledging the positive effects of yoga on alleviating psychological disorders and stress-related diseases (Cramer et al., 2013; Pascoe et al., 2017; Breedvelt et al., 2019) as well as promoting mental and physical health (Büssing et al., 2012; Gothe and McAuley, 2015; Hendriks et al., 2017). However, high heterogeneity among yoga practices and poor methodological quality have limited the generalizability of these findings. Apart from this, previous yoga research has exhibited two major shortcomings. First, yoga incorporates diverse components, which have only insufficiently been investigated and differentiated in the past (Gard et al., 2014; Schmalzl et al., 2015). We know neither how each single component of yoga works nor what impact specific combinations of these components have. Second, the ethical component of yoga has frequently been neglected in the past. In a bibliometric analysis, only 10% of yoga studies explicitly incorporated lectures on yoga philosophy or ethics (Cramer et al., 2014). Yet, traditional yoga experts have advocated that yoga should be practiced in its entirety, including its ethical aspects (Varambally and Gangadhar, 2016).

Traditional yoga dates back over 5,000 years and was originally understood in a much broader sense than is common in Western contemporary settings. Indeed, classical, or *raja* yoga, as outlined by Patanjali (author of the Yoga Sutras; Bryant, 2015), was primarily a system of meditation. Patanjali described the aim of yoga as the stilling of the fluctuations and changing states of the mind that cause suffering (“Yogah chitta vrtti nirodhah,” Yoga Sutras, Chapter 1, Verse 2; Patanjali between 600 BC and 200 AC; Bryant, 2015). The aspiring practitioner could reach this still state through the practice of the eight limbs of yoga, also referred to as the eight-fold yoga path (*ashtanga* yoga). This path comprises the following practices: *yamas* (universal ethics), *niyamas* (individual ethics), *asana* (physical posture), *pranayama* (breath control), *pratyahara* (withdrawal of the senses), *dharana* (concentration), *dhyana* (meditation), and *samadhi* (full meditative absorption; Feuerstein, 2012; Bryant, 2015).

Recent theoretical proposals have taken into account this multitude of yoga practices (Gard et al., 2014; Schmalzl et al., 2015; Sullivan et al., 2017). All of these proposals strongly encourage the empirical investigation of the specific components of yoga and suggest conducting longitudinal, comparative, or dismantling studies. Yet, the multitude of possible “active ingredients” in yoga makes the investigation of its components challenging. Modern styles of yoga have diverted considerably from the “classic” eight-fold yoga path and often reduced its inherent multifariousness. Many styles focus primarily on postures and breathing practices, for example, Ashtanga, Iyengar, or Hatha Yoga in general. There are also yoga styles that comprise mainly breathing practices (e.g., Surdashan Kriya), or meditation (e.g., Sahaja Yoga). Some place particular attention to include mantras, chanting and music (e.g., Kundalini Yoga). For an overview on different yoga styles, see McCrary (2013). Just as multifaceted as yoga styles are scientifically investigated yoga interventions. Cramer et al. (2016) reviewed studies investigating 52 different yoga styles and concluded that the proportion of positive outcomes did not vary across styles. However, this analysis did not dismantle the different components of yoga styles.

A recent review summarized comparative studies and meta-analyses of the effects of yoga components (Matko et al., 2021a). The authors concluded that although most of the treatments compared were equal in length, outcomes were better for those that combined several elements of yoga practice. Frequently, combining yoga postures with breathing practices, meditation, or ethical education enhanced the effectiveness of the intervention. This finding was also reported in several meta-analyses (e.g., Gong et al., 2015; Wu et al., 2019). But these studies were very heterogeneous and many findings were inconclusive. Even if combined interventions were mostly more effective, it is not known which specific benefit could be attributed to which component of these interventions.

Often, studies compared rather complex interventions with each other without isolating specific parts of the interventions. For example, Quach et al. (2016) compared a physical yoga program to a mindfulness meditation program, but both programs included breathing exercises and group discussions. Granath et al. (2006) contrasted a physical yoga intervention with a cognitive behavioral therapy program; however, both interventions included psychoeducation and relaxation. Gorvine et al. (2019) compared physical yoga to a meditation program composed of different meditation techniques. Yet, as we know, different meditation techniques produce different effects (Fox et al., 2016; Kropp and Sedlmeier, 2019). Employing such study designs definitely yields interesting insights but makes it hard to uncover how each specific component of these complex programs works. In addition, we found only one study that explicitly examined the effects of adding an ethical education component to a complex yoga intervention (Smith et al., 2011).

Thus, it seems advisable for yoga research to evaluate yoga in its entirety and investigate the specific mechanisms and benefits of each yoga component. There have been repeated calls in this regard to fully understand the underlying mechanisms of yoga (Sherman, 2012; McCall, 2013; Riley and Park, 2015). The investigation of yoga components would facilitate the development of more targeted and efficient programs tailored to the specific needs of respective clinical or healthy populations (Gard et al., 2012; Schmalzl et al., 2015). To date, there have been only a few investigations into this matter. One particularly under researched area of interest is the incorporation of yoga ethics into intervention studies. Furthermore, it remains unclear whether there are specific combinations of yoga practices that yield better effects than others do. Hence, the present study was aimed at bridging this gap. Employing a single-case multiple-baseline design, we compared the relative benefits of adding ethical education and/or physical postures to a simple mantra meditation intervention.

Almost all meta-analyses and theoretical proposals on yoga criticize the lack of methodological accuracy in previous yoga studies. Longitudinal and dismantling studies have been proposed as an effective means to (1) study mechanisms of mind–body practices/yoga, and (2) provide optimal control groups (Kinser and Robins, 2013). In addition, conventional research designs reach their limits in yoga and meditation research as there is no overarching theory that would guide systematic investigations, group comparisons cannot capture specific changes over time or individual differences properly, and purely quantitative approaches are limited with regard to participants' individual perceptions of change (Schmalzl et al., 2015; Sedlmeier et al., 2016; Lundh, 2020). Consequently, employing mixed-methods or repeated-measures designs might be more helpful in this respect. Recently, there has been a rise in elaborate studies using daily assessments before, during, and/or after an intervention in experience sampling or single-case research designs (May et al., 2014; Shoham et al., 2017; Lindsay et al., 2018; Singh et al., 2019; Bai et al., 2020). The present study is in line with these research efforts.

In experimental single-case research designs (Barlow et al., 2009) dependent variables are measured very frequently over extended periods of time. Accordingly, they allow for a more detailed examination of individual responses and processes of change and are, thus, more suitable for explorative research questions such as ours. Several authors have suggested that individual differences might tremendously influence the effects of meditation and yoga (Hölzel et al., 2011; Gard et al., 2014; Lippelt et al., 2014). Furthermore, participants are often treated as collaborators rather than “subjects” enabling a closer cooperation and quantitative as well as qualitative insights facilitating a mixed-methods approach. Hence, single-case research does not necessarily require participant blinding. Multiple-baseline designs consist of an A phase (baseline) and a B phase (treatment), but the length of the A phase is varied across different participants creating a staggered introduction of the intervention and making possible horizontal and vertical comparisons (Ferron et al., 2014). In this design, randomization happens over time instead over people producing strong internal validity. If there is a strong contingency between the treatment and a certain effect, irrespective of when the treatment starts, this will be a solid argument for the causal role and effectiveness of the treatment.

This study was part of a project evaluating a new mind–body program called Meditation-Based Lifestyle Modification (MBLM; Bringmann et al., 2020). This holistic program encourages and empowers participants to adopt a beneficial lifestyle in order to experience sustained eudaemonic well-being, mental health, and human flourishing. Meditation-Based Lifestyle Modification is based on the eight-fold yoga path and covers three main domains that correspond, in short, to (1) yoga's ethical education, (2) postures and breathing practices, and (3) meditation. We describe them in more detail in the Method section. The type of meditation taught is mantra meditation. Previous reviews and meta-analyses substantiated the positive effects of mantra meditation (Sedlmeier et al., 2012; Lynch et al., 2018). However, they also criticized the poor methodological quality of most studies on mantra meditation and recommended conducting higher quality research into this topic.

We employed MBLM as a test bed for our research. We dismantled the MBLM program and investigated different combinations of its components. At the same time, we evaluated MBLM's efficacy in a healthy population. Although it has been designed as a mind–body therapy for patients with mental disorders, it might be beneficial for preventive purposes, too. Comparable preventive effects have been observed for Mindfulness-Based Stress Reduction (MBSR), which was originally developed for patients suffering from chronic pain (Kabat-Zinn, 1982). Meanwhile, it has become a widespread and widely researched intervention for all kinds of conditions (Grossman et al., 2004). Moreover, yoga was initially designed as a spiritual path for healthy individuals (Feuerstein, 2012). Therefore, we would expect positive outcomes for an intervention that incorporates as many yoga components as MBLM.

Both yoga theory and research literature suggest that combined interventions should be more effective than simple interventions. Yet, research findings were inconclusive on determining what specific combinations were best for what purpose (Matko et al., 2021a). Consequently, we chose an additive design and designed four conditions (see below). From theory, we would expect a small effect for the meditation alone condition, a larger effect for the two conditions including meditation and another component, and the largest effect for the full MBLM program. Conversely, it might be equally reasonable to expect a specific combination of components to be more effective than the full program. The investigation of the ethical component in this study is of particular relevance, as it might have an even bigger impact on participants than physical yoga (Smith et al., 2011). To our knowledge, no other study has contrasted all of these combinations in a comparative study. Moreover, no other study has employed a multiple-baseline design for this purpose. As this field of investigation is relatively new, we combined quantitative and qualitative methods to explore inter-individual differences and differences between conditions.

The present study is part of a larger project in which we measured a wide range of dependent variables commonly associated with the effects of yoga and meditation. Selection of variables was based on theoretical considerations and suggestions found in existing literature (Hölzel et al., 2011; Gard et al., 2014). For this paper, we decided to focus on the most commonly investigated outcomes in the yoga literature: well-being, stress, and life satisfaction. Findings on these variables were not always unequivocal, especially for stress and life satisfaction. Following our considerations above, we would expect favorable effects on all outcome variables, but specifically for the combined conditions. Nevertheless, the present study is exploratory in nature. Thus, we refrained from formulating predefined hypotheses and focused on two central research questions instead. First, what are the incremental effects of ethical education and physical yoga on mantra meditation? Second, what combinations of components are particularly effective? Is more or is less more?

## Methods

### Procedure

This study employed a multiple-baseline design with a priori determined phase lengths. During baseline, participants engaged in their usual daily activities and received no treatment. We randomized participants across three baseline lengths (7, 14, or 21 days) and four treatment conditions. The conditions were mantra meditation alone (MA), mantra meditation plus physical yoga (MY), mantra meditation plus ethical education (ME), and mantra meditation plus physical yoga and ethical education (MYE). Each treatment lasted 8 weeks and participants started according to their baseline condition. The overall study duration varied across participants, ranging from 9 to 11 weeks. Treatments were run on Thursdays (MYE 9:00 to 12:00 a.m.; MA 1:00 to 2:00 p.m.) and Fridays (ME 9:00 to 11:15 a.m.; MY 12:15 a.m. to 2:00 p.m.).

**Figure 1:**
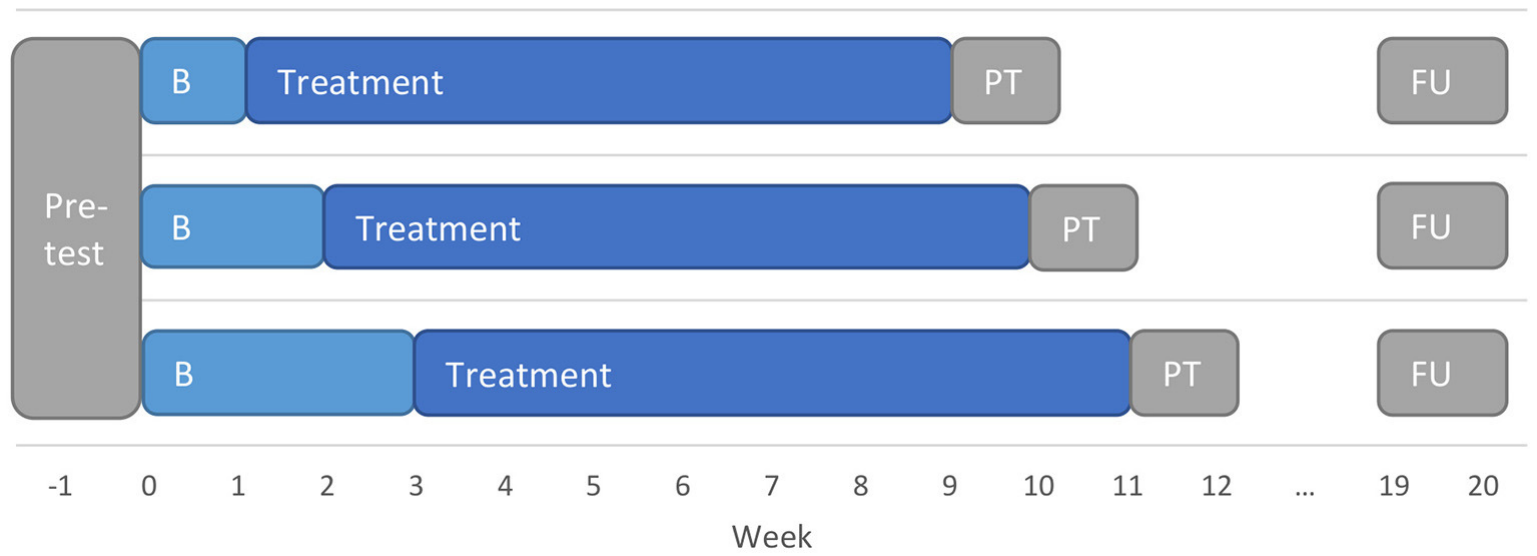
Multiple-baseline design employed in the present study. B, baseline; FU, follow-up; PT, Posttest.

All measurements were taken online. Participants completed an extensive battery of questionnaires (see Supplementary Material A—Table A1) during pretest in the week before the baseline measurements commenced. All participants started their baseline measurements on the same day and received daily online questionnaires throughout their entire baseline and treatment phases. After the treatment had ended, participants completed another battery of questionnaires during posttest. Follow-up measures were taken 8 weeks and 12 months after posttest. Figure 1 depicts the study design.

### Participants

We recruited participants via the central experiment server and the university sport mailing list of the Dresden University of Technology and through flyers and handbills distributed in Dresden. All those interested had to complete a short online screening survey. Two hundred thirty-six people completed the screening survey, of whom 128 did not meet our inclusion/exclusion criteria and 51 declined to participate. Participants had to be older than 18 years and had to ensure they had daily access to web-enabled devices. Exclusion criteria were pre-existing psychiatric conditions, acute psychological issues, or a regular yoga or meditation practice during the last 6 months. Those meeting our criteria were invited to an information event, which was led by KM and HCB. During this event, we fully disclosed the nature of the study to participants, but emphasized that this study was exploratory and we did not know what effects the different conditions might have. Participation was voluntarily and all participants provided written consent to participate in the study. They received no financial or other compensation for their participation in the study, but they had the opportunity to win one of ten €50 gift coupons at the completion of the study. The institutional review board of the Chemnitz University of Technology approved the experimental protocol.

**Figure 2:**
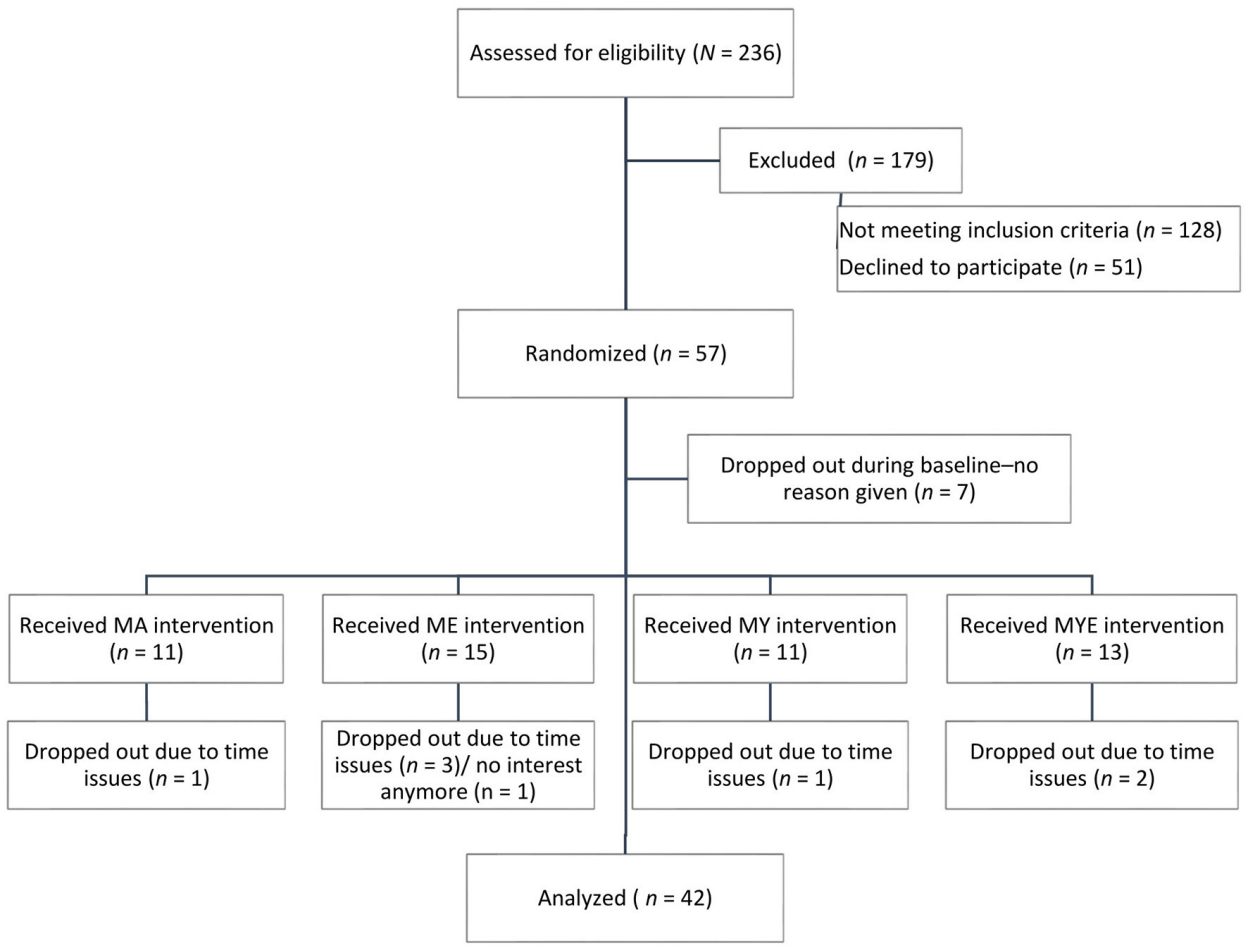
CONSORT flow chart of participants in the study.

Fifty-seven meditation-naïve participants were randomized to one of 12 subgroups employing simple random sampling without replacement in Excel. Prior to randomization, each subgroup received a number and participants were allowed to indicate a preferred day (Thursday/Friday) without knowing which treatment took place on which day. We used these indications to split the sample into two equal blocks (Thursday/Friday), tossing a coin if participants had not indicated a preferred day. Then, we generated random numbers for each participant within each block and assigned them to one subgroup in ascending order. Their treatment condition was revealed to them directly after randomization, but were not allowed to switch to another group. Seven participants dropped out during the baseline phase before the intervention started and provided no reason for dropping out (see Figure 2). Eight participants dropped out during the intervention, mostly because of time issues. Although there was some attrition toward the end of the data collection period (see below), but not during posttest or follow-up, we decided to include all remaining participants in our analysis. Single-case research designs allow for a much closer examination of each case and the statistical methods we employed for data analysis are relatively robust against missing data. The final sample consisted of 42 participants (83.3% female, mean age 26.62 years, *SD* = 8.37). Sociodemographic data differed slightly across conditions (see Table 1) and was, therefore, statistically controlled in our statistical analyses.

**Table 1:** Sociodemographic data of participants in each condition.

**Variable**	**Condition**				**Total**
	**MA**	**ME**	**MY**	**MYE**	
*n*	10	11	10	11	42
Gender (% female)	90.0	72.7	90.0	81.8	83.3
Age (years)					
*M* (*SD*)	29.00!!! (10.40)	25.09!!! (5.74)	27.60!!! (12.32)	25.09!!! (2.95)	26.62!!! (8.37)
Range	22–57	19–36	18–61	19–30	18–61
Occupation					
% students	80.0	72.7	90.0	100.0	85.7
% employed	20.0	27.3	10.0	0.0	14.3

*MA, Mantra meditation; ME, meditation and ethical education; MY, meditation and physical yoga; MYE, meditation, physical yoga, and ethical education*.

### Treatment: MBLM and Its Components

Weekly training sessions were dedicated to practicing together and discussing emergent questions or difficulties. All treatments were jointly led by KM and HCB, who are both experienced meditation teachers. HCB is an accredited psychiatrist and psychotherapist. KM is a psychologist and certified yoga instructor with 700 h of teacher training and 6 years of teaching experience. All treatments took place in the same seminar room that we rented for the study.

The length of the weekly sessions varied across conditions as each component had different time requirements: 25 min for meditation, 50 min for physical yoga, 75 min for ethical education, and at least 20 min for group sharing, plus time for breaks as required. The overall duration for each condition was as follows: MA = 60 min, MY = 105 min, ME = 135 min, MYE = 180 min. Each training session started with a discussion of participants' experiences and practice at home since the previous session and ended with another group sharing. All three components are explained in more detail in Supplementary Material A—Section A2.

All four conditions involved learning to meditate using mantra meditation. The key practice in this form of mantra meditation is silently repeating the chosen mantra while letting all other thoughts pass by and letting the breath flow naturally. Each weekly session in every condition included a 25-min silent (i.e., non-guided) mantra meditation practice. During the physical yoga practice, each class started with approximately 10 min of breathing exercises, followed by 30 min of postures and dynamic exercises, and concluded with a 10-min guided relaxation (see Table 2; for more detailed information on yoga practices, see, e.g., Iyengar, 2009; Stephens, 2011).

**Table 2:** Set of physical yoga and breathing exercises taught in the physical yoga component including their sanskrit names, short descriptions, and proposed health benefits.

**Yoga exercise**	**Sanskrit name**	**Description and health benefits**
**Breathing techniques (** ***pranayama*** **)**		
Deep yogic breathing	Dirgha Pranayama	Deeply inhaling and exhaling into abdomen, chest and clavicular region, learning to use full breathing capacity
Victorious breath	Ujjayi Pranayama	“Ocean breath”: Slightly contracting the throat while breathing, learning to inhale and exhale more slowly, more fully, and with more control
Dynamic breathing	Unknown	Standing and taking deep breaths while simultaneously moving the arms and the body in the rhythm of breathing, learning to match movement and breathing, activating effect
**Postures (** ***asana*** **) and dynamic exercises (** ***vinyasa*** **)**		
Sun salutation	Surya Namaskar	Set of 12 postures practiced subsequently in the rhythm of breathing; engages and warms up the whole body
Mountain pose	Tadasana	Gain sense of stable and good posture
Tree pose	Vrksasana	Improves balance
Eagle pose	Garudasana	Preparatory exercise: rolling shoulders; stretches area between the shoulders, strengthens legs, improves balance
Warrior pose II	Virabhadrasana II	Strengthens and stretches ankles, calves, and thighs, opens hips
Triangle pose	Trikonasana	Stretches and strengthens sides of the body
Wide-legged forward bend with clasp pose	Prasarita Padottanasana C	Opens shoulders and chest, stretches legs and the spine
Knees-to-chest pose	Apanasana	Relaxes back and neck, improves digestion
Supine spinal twist pose	Supta Matsyendrasana	Stretches abdomen and lower back, relaxes shoulders and back
Legs up the wall pose	Viparita Karani	Mild inversion pose, restorative
Boat pose	Navasana	Strengthens abdominal muscles
Cobra pose	Bhujangasana	Strengthens upper back, improves digestion
Child's pose	Balasana	Relaxes upper back, neck, and arms
Bound Angle Pose	Baddha Konasana	Opens hips
Half-spinal-twist pose	Ardha Matsyendrasana	Relaxes spine and neck, opens chest, tones waist
Deep relaxation in corpse pose	Savasana	Restorative, different relaxation techniques (autosuggestion, body scan)

Ethical education followed the protocol developed for the MBLM mind–body program (Bringmann et al., 2020). Each week, we introduced one of the 10 *yamas* and *niyamas*, with the last three *niyamas* being grouped together for time reasons into one topic called “transcendence.” After we introduced each topic, we invited participants to discuss its application and relevance for their daily lives and engage in related mindful living activities (see Table 3) during the following week. In the next session, they shared and reflected upon their experiences. Participants received handouts for each treatment component including detailed information and instructions for practicing at home. We asked all participants to practice their respective treatment practices daily, that is, 20 min of mantra meditation, 20 min of yoga exercises, and/or mindful living activities.

**Table 3:** Topics of the ethical education component with corresponding week(s) they were taught in the study, sanskrit names, and sample mindful living exercises.

**Week**	**Topic**	**Sanskrit name**	**Sample mindful living exercises**
***Yamas*** **—universal ethics/right living with others**			
1 and 9	Non-violence	Ahimsa	Practice praising instead of criticizing (also of myself) !!! Practice respecting my boundaries (e.g., taking breaks)
2 and 10	Truthfulness	Satya	Write down how I really think and feel !!! Practice being truthful instead of “nice”
3	Non-stealing	Asteya	Recognize inner and outer abundance in my life !!! Practice giving when I receive something
4	Self-restraint	Brahmacharya	Enjoy eating/working/watching TV before excess or inertia sets in
5	Non-hoarding	Aparigraha	Clear things out that I don't need !!! Recognize expectations I have concerning myself and others
***Niyamas*** **—individual ethics/right living with yourself**			
6	Cleanliness	Sauca	Practice bodily cleansing (e.g., intermittent fasting) !!! Recognize and enjoy moments of purity
7	Contentment	Santosha	Practice being thankful for things that happened today !!! Refrain from chasing or avoiding specific things I like/dislike
8	Transcendence	Tapas (self-discipline) !!! Svadhyaya (self-study) !!! Ishwara pranidana!!! (devotion)	Practice faculty of discrimination (“Is this conductive to my goals?”) !!! Read a spiritual text!!! Try to connect to the miracles of life

### Measures

Instruments for daily/weekly measures had to be suited to experimental single-case designs in that they had to be precise, relatively short, and sensitive to changes while not exhibiting floor or ceiling effects. We carried out extensive preparatory work and a pilot study (Quasten, 2019) to test and finalize our selection of instruments. All questionnaires were programmed and implemented with SoSci Survey (Leiner, 2019) and made available to participants on www.soscisurvey.com. Data were collected between 21 March 2019 and 31 July 2019.

#### Well-Being

Participants' daily well-being was measured with the very short and economical World Health Organization Well-Being Index (WHO-5; World Health Organization, 1998). The WHO-5 is a psychometrically sound self-report measure with high internal consistency and high convergent validity (Brähler et al., 2007). It consists of five items that were rated on a 6-point Likert scale. High scores represent a high state of well-being. As we collected well-being daily in the present study, we adapted the time frame of this measure to “the last 24 h.”

#### Stress

The Perceived Stress Scale (Cohen and Williamson, 1988) is a widely used self-report measure that we employed weekly in the present study to measure stress. It intends to capture the degree to which people perceive situations in their life as excessively stressful relative to their ability to cope. Respondents rated each of the 10 items on a 5-point Likert scale of 1 (*never*) to 5 (*very often*). It has shown good internal consistency (α = 0.78) and moderate convergent validity.

#### Life Satisfaction

The Satisfaction with Life Scale (SWLS; Diener et al., 1985) has been extensively used as a measure of the life satisfaction component of subjective well-being. We used it in the present study during pre- and post-testing. The SWLS is a very short self-report measure with five items that are rated on a 7-point Likert scale of 1 (*strongly disagree*) to 7 (*strongly agree*). Internal consistency of the scale is high (α = 0.92).

#### Daily Practice

With the beginning of the treatment phase, we asked participants to track their home practice in the daily questionnaire. All participants had to supply information on the length of their daily meditation practice (“How many minutes did you meditate today? Fill in ‘0’ if you did not practice today.”) as well as the time of day they practiced. Furthermore, they were asked to rate their experiences with this day's meditation practice on a 5-point polarity profile. They were presented with three items to measure (a) experienced difficulty/ease (“Meditating was very difficult … very easy”), (b) wakefulness (“I was feeling sleepy … awake), and (c) relaxation (“I was feeling very restless … very relaxed”). Participants in conditions including physical yoga practice were asked to provide information on their yoga practice in a similar manner. We added one more item to assess experienced coherence of breath during yoga practice (“The practice and my breath were non-coherent … coherent”). Participants in ethical education conditions were asked only two questions, about (a) engagement in ethical practice (“Did you engage in any of the mindful activities today?”) and (b) experienced difficulty of the current topic of ethical education.

#### Course Satisfaction

The Client Satisfaction Questionnaire (CSQ-8; Attkisson and Zwick, 1982) was developed to assess global client satisfaction along a single dimension in clinical settings. We used it in this study during posttest to determine participants' satisfaction with the course they completed. The CSQ-8 has eight items that are rated using a 4-point Likert scale. It is considered a reliable (α = 0.92) and valid instrument.

#### Adverse Events

The posttest included a list of 70 possible adverse events or extraordinary experiences associated with meditation or yoga practice. We gathered this list from several publications on adverse effects of meditation and yoga (Matsushita and Oka, 2015; Cebolla et al., 2017; Lindahl et al., 2017) and categorized all events and experiences into 10 clusters of related symptoms: neurological, somatic, pain, cognitive, emotional, motivational, changes in necessities, difficulties in life, compulsive meditation, and altered states of consciousness (see Supplementary Material A—Table A4). Participants were instructed to mark all events and sensations they had experienced during the treatment phase and to rate their severity (mild, moderate, severe) and duration in days (1–2, 3–6, 7–13, 14–20, ≥21), respectively.

#### Special Occurrences

Participants had the opportunity to describe any special events that occurred throughout their day in a free text item in the daily questionnaires.

### Data Analysis

Single-case data are usually analyzed using multiple approaches, the most common being visual inspection of dependent-variable-by-time plots (Gage and Lewis, 2013; Lane and Gast, 2014). There are multiple ways how these data can be analyzed statistically, with multifaceted proposals and ongoing debates concerning this issue (Burns, 2012; Evans et al., 2014; Shadish, 2014; Machalicek and Horner, 2018). Various effect size estimates have been proposed, each with their individual advantages and disadvantages (Parker et al., 2011a; Tarlow, 2017; Pustejovsky, 2019). We analyzed data in three ways—by visual inspection, calculating effect sizes using Tau-*U*, and multilevel modeling. All methods are described below. If all three analyses converged, this would provide strong evidence for our findings. In addition, we enriched our quantitative analyses with qualitative findings, where appropriate, to explore selected single cases and possible reasons for inter-individual differences.

Statistical analyses on the incremental effects of the four conditions were exploratory in nature. We repeated two coding schemes using different dummy variables. To investigate whether there were any general differences between the four conditions, we used three dummy variables to code the four conditions (condition model). We used this model to estimate the overall explanatory power of the dummy variables using the *anova*-function in R. To examine whether there were any differences regarding the inclusion of different program components, we prepared two other dummy variables. These coded the presence vs. absence of ethical education or physical yoga (0 = *without component*, 1 = *with component*) in the respective condition (component model). As our four treatments differed in session length and demographic factors, we included individual practice time, age, gender, occupation, and baseline length in both models to control their influence statistically. To estimate individual practice time, we calculated sum scores of the reported length of each practice participants engaged with at home. For ethical practice, we multiplied participants' entries (1/0) by 20 min to get a comparable estimate of practice duration. As we expected combined interventions to have stronger effects than the simple meditation intervention, we applied one-tailed tests of significance by dividing the resulting *p* levels by two. We considered *p* < 0.05 to be statistically significant.

All statistical analyses were performed using R 3.6.3 (R Core Team, 2020). Plots were generated with the statistical packages lattice (Sarkar, 2008) and ggpubr (Kassambara, 2020). Tau-*U* estimates were calculated and analyzed using the package scan (Wilbert and Lueke, 2019), and multilevel models were conducted using the package nlme (Pinheiro et al., 2020). Proportion of explained variance in multilevel models was calculated using the R-based online application mimosa (Titz, 2020). All scripts and data that support the results can be found at osf.io/n7y64/.

#### Visual Analysis

We kept our visual analysis relatively simple for pragmatic reasons as our sample was exceptionally large for an experimental single-case study. We used the R packages scan and lattice to generate individual dependent-variable-by-time plots with according level and trend lines. Following common visual analysis standards (Kratochwill et al., 2010), we then assessed whether there were perceivable trends in the baseline or the treatment phase, and whether there were differences between the means and the variability of data in each phase. Furthermore, we analyzed the immediacy of the effect after the onset of the treatment and the consistency of data patterns across individuals. Finally, we compared all individuals in one condition to individuals in the other conditions to see whether the observed patterns differed between conditions.

#### Tau-*U*

To provide a nuanced measure of phase non-overlap we calculated Tau-*U*. Parker et al. (2011b) initially proposed Tau-*U* as a non-parametric estimate of effect size in single-case research designs that allows controlling trends observed in both phases. Tau-*U* is a family of non-parametric rank correlation indices that, as such, are relatively robust to autocorrelation and have shown good statistical power. In this study, we calculated Tau-*U* coefficients for each participant and each dependent variable. We assumed trends in the data to be theoretically probable, both in response to repeatedly filling out questionnaires in the baseline phase and in the form of a continuous improvement in the treatment phase. Therefore, we corrected trends in both phases if they were statistically significant, larger than 0.40, or visually prominent. Accordingly, we chose and reported corrected effect size estimates for these individuals (Tau-*U*_A vs. B−Trend A_, Tau-*U*_A vs. B+Trend B_, or Tau-*U*_A vs. B+Trend B−Trend A_). If no trends were evident, we reported Tau-*U*_A vs. B_. We applied the interpretative benchmarks provided by Solomon et al. (2015) where an effect size of less than 0.28 indicates a small effect; 0.29–0.47 a moderate effect; 0.48–0.57 a large effect; and 0.58 or above a very large effect.

We explored possible differences between conditions by first generating and comparing boxplots of Tau-*U*s for the four conditions. Second, we conducted two multiple regression analyses predicting Tau-*U* estimates by the different dummy variables described above. Analyzing the effects of condition or component on effect size estimates resembles cross-level interactions in multilevel modeling.

#### Multilevel Modeling

Multilevel modeling (also known as hierarchical linear modeling) is a powerful tool for modeling correlated data in which observations are nested within individuals and for examining both individual change and group differences (Dedrick et al., 2009; Hox, 2010). It has been proposed as a suitable method for analyzing multiple-baseline data (Ferron et al., 2009). In this study, we modeled changes over time within each individual on one level and differences between individuals on a second level. Prior to all analyses, we standardized all variables to obtain standardized regression coefficients and reliable interaction terms.

For each dependent variable, we estimated several models with increasing levels of complexity. However, as we were primarily interested in the cross-level interactions, we report only the final models. The full estimation procedure can be found in Supplementary Material B (Tables B1, B2). We modeled a cross-level interaction between time and dummy variables to determine whether any condition or component had an incremental beneficial effect on participants in this study. The following equation shows the final component model:

$$\begin{array}{ll} y_{ij} & =\;\gamma_{00}+\;\gamma_{01}{ethical\;education}_{j}+\;\gamma_{02}{physical\;yoga}_{j}\\ + & \;\gamma_{03}age_{j}+\;\gamma_{04}gender_{j}+\;\gamma_{05}{baseline\;length}_{j}+\;\gamma_{10}time_{ij}\\ + & \;\gamma_{11}{ethical\;education}_{j}\;*\;time_{ij}+\gamma_{12}{physical\;yoga}_{j}\;*\;{time}_{ij}\\ + & \;u_{0j}+\;u_{1j}time_{ij}+\;r_{ij} \end{array}$$

where *y*_*ij*_ refers to the dependent variable, all γ variables refer to fixed effects, and all *u* and *r* variables refer to random effects.

Time was a contrast-coded Level 1 variable representing the expected slope of change that occurred from baseline to treatment phase. It was coded with zero for the baseline phase as we expected no systematic change in this phase, and a logarithmic trend starting at the beginning of the treatment phase. We applied the logarithmic curve as this is a type of growth commonly observed in psychology (Jones et al., 2005), and it provides a better conceptual fit than a linear trend. If we observed substantial variation in individual slopes during visual inspection, we modeled time as a random slope. Furthermore, we applied one-tailed tests of significance to the time variable as we expected all treatments to exert a positive effect on our participants.

Data were screened and corrected for (illegitimate) outliers due to data-entry errors. Other (legitimate) outliers were hard to identify. Hence, we treated them conservatively by not excluding them. Following a proposal of Nakagawa and Schielzeth (2013), we used two effect size estimates to assess the proportion of explained variance in each model, namely, marginal *R*^2^ (variance explained by fixed factors), and conditional *R*^2^ (variance explained by both fixed and random factors). All models were estimated using the restricted maximum likelihood estimation procedure.

#### Missing Data

Because of the admittedly high response load with daily questionnaires over a period of 71–85 days, we did have some missing data in the present study, specifically toward the end of the data collection period. Some data points were missing because some participants simply forgot to respond to the questionnaire on some days, or because a few participants reported (during class) stressful life events that kept them from responding. Mean amount of missing data across participants was 18.5% (range 2.8–45.9%). We performed all analyses with the data available bearing in mind the limitations of this approach (Peng and Chen, 2021). Only one participant failed to respond at posttest and follow-up and another one failed to respond at follow-up. We excluded these two participants from the analysis of life satisfaction.

## Results

In this section, we first report on participants' adherence to our treatment. Then, we present the results on our three main outcome variables: well-being, perceived stress, and life satisfaction. For the two continuously measured variables, we first present individual plots for each participant and report the results of our visual inspection. Second, we report on our statistical analyses of these variables employing Tau-*U* effect size estimates and multilevel modeling. In the final part of the Results section, we explore possible moderator variables that might help explain the effects found for our main outcomes. These moderator variables are course satisfaction, adverse, or extraordinary events experienced during the treatment, and subjective experiences with the daily practice.

### Adherence

First, we looked at whether participants actually engaged in their respective daily home practice. Compared to all other conditions, participants in the meditation-only condition reported significantly higher daily meditation practice durations, *M*_MA_ = 18.2, *SD* = 9.7; *M*_ME_ = 14.7, *SD* = 9.3; *M*_MY_ = 14.0, *SD* = 9.8; *M*_MYE_ = 13.1, *SD* = 10.2; *F*_(3, 1,908)_ = 23.45, *p* < 0.001. This might be because, in contrast to the other conditions, this was the only home practice participants were supposed to engage with. We plotted the engagement in all three home practices over time and examined the respective plots (see Supplementary Material C—Figures C1–C3). For meditation practice, we observed a decline in practice duration across all conditions toward the end of the study. Remarkably, participants in the MYE condition reported higher average practice duration/frequency in Hatha yoga and mindful living activities, compared to participants in the ME or MY conditions, yoga: *M*_MYE_ = 15.7, *SD* = 13.1; *M*_MY_ = 12.6, *SD* = 12.2; *t*_(959)_ = −3.81, *p* < 0.001, and ethics: *M*_MYE_ = 0.74, *SD* = 0.44; *M*_ME_ = 0.68, *SD* = 0.47; *t*_(1,046)_ = −2.18, *p* = 0.030. However, practice times may be underestimates, as participants may have engaged in home practice on days for which they did not complete the daily questionnaire.

Course adherence was moderate to high. Apart from four participants who attended only one, two, or three group sessions but consistently practiced at home, the majority of participants attended at least six of the eight group sessions. Adherence was a bit higher in conditions that involved ethical education (*M*_ME_ = 6.09; *M*_MYE_ = 6.64) than in the other two conditions (*M*_MA_ = 5.50; *M*_MY_ = 5.20).

### Well-Being

#### Visual Analysis

**Figure 3:**
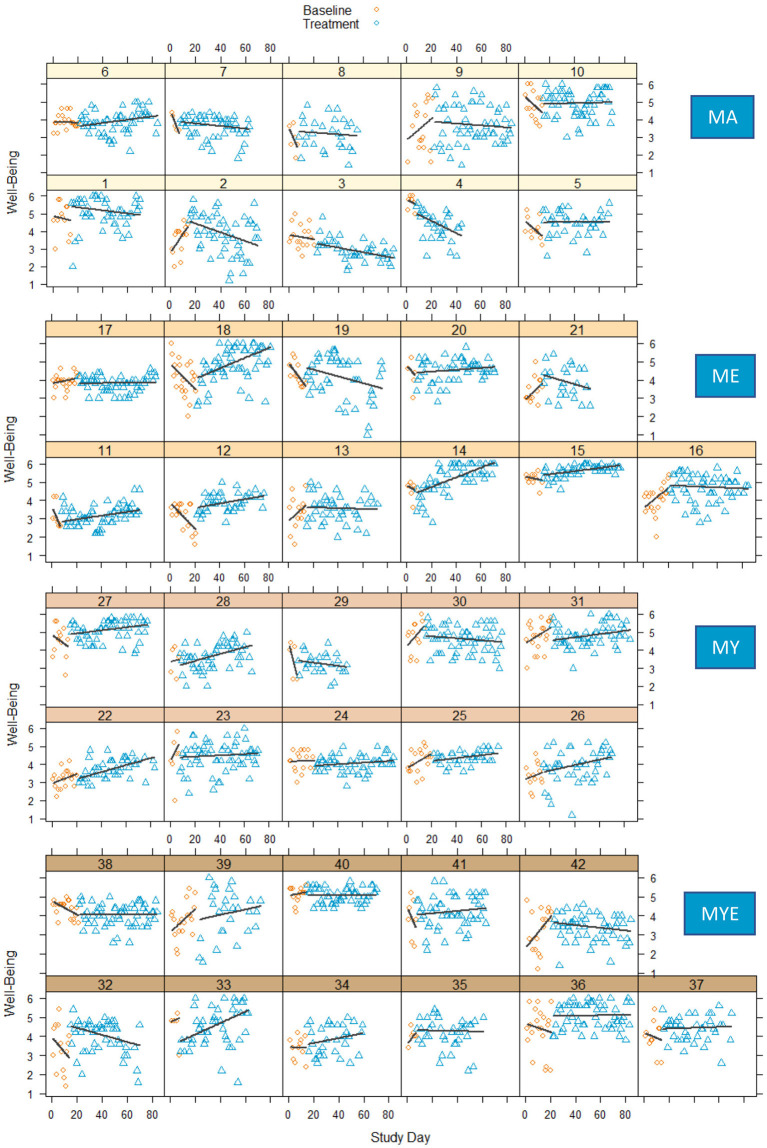
Well-Being scores in four conditions during baseline and treatment phases for each participant with regression lines for each phase. MA, Mantra meditation only; ME, meditation and ethical education; MY, meditation and physical yoga; MYE, meditation, physical yoga, and ethical education.

Figure 3 depicts the well-being scores of each participant over the course of time. It is subdivided into four plots, one for each condition. As can be seen from this figure, well-being scores show strong fluctuation and variation over time and the amount of daily fluctuation varies inter-individually. Some points in this figure stand out as days with especially low well-being. Most of these days correspond to life events participants experienced as very challenging, for example, exams, illnesses, or a separation, and reported during the weekly sessions.

For most participants, the baseline phase cannot considered to be stable. Around half of the participants showed a decline in their well-being scores during the baseline phase and a third showed an increase, the reason for this finding being unclear. Comparing both phases, some participants showed no observable changes in level and/or slope (e.g., Participants 9, 23, 31, 40), whereas most profited from the treatment, some more obviously (e.g., Participants 14, 15, 22, 36) than others (e.g., Participants 11, 26, 27, 28). Strong positive effects seem to be present predominantly in the ME condition. On the other hand, some participants exhibited a decline in well-being over the course of time, especially toward the end of the treatment. This might indicate an increasing fatigue with the intervention, which might be particularly true for those participants who prematurely stopped responding to the daily questionnaires (e.g., Participants 4, 21, 29). Yet, the decline was most pronounced in the group of participants who were practicing mantra meditation only (e.g., Participants 1–4).

For most participants, well-being increased gradually either from the beginning of the treatment or after a small delay of 1 or 2 weeks. Quite a few participants reported having mastered the meditation technique after initial difficulties at 2 weeks after the beginning of the treatment. After a few more weeks, however, participants reported getting bored or feeling stuck with meditating. While some of them found ways to revive their motivation or find deeper meaning in meditating, others resigned, or tried to uphold their meditation practice without connecting it to a deeper meaning. We observed the latter more frequently in the MA condition and less frequently in the other conditions. These qualitative findings correspond to our analysis of experienced meditation difficulty over time, which we report at the end of the Results section. Intriguingly, it seems that the experiences during the process of learning meditation were closely related to daily well-being.

Overall, these results strengthen the impression that people respond quite differently to meditation interventions. There seem to be discernible differences between the four treatment conditions, too, indicating a negative effect of the meditation-only intervention. To further explore and validate our visual analysis we conducted two distinct statistical analyses.

#### Statistical Analysis

##### Tau-U

The Tau-*U* statistic was calculated to assess the effect size of the intervention for each participant (see Supplementary Material B—Table B3). Most effect size estimates ranged from 0.20 to 0.40, indicating a small to moderate improvement of well-being for the majority of participants. We observed the largest positive effect sizes (0.42–0.46) for Participants 14, 15, and 22. Three participants (3, 4, and 38) had a substantially lower well-being following the treatment (−0.30 to −0.51).These results correspond to our visual analysis.

Next, we looked at potential explanations for the strong negative effects. Participant 3 (MA) had an especially hard time trying to learn mantra meditation. She almost always rated her meditations as being very hard. Participant 38 (MYE) deeply appreciated her course but reported having elevated levels of stress due to beginning work on her master's thesis, which coincided with the beginning of the treatment. We had considered excluding Participant 4 (MA) from the analysis as she attended only the first two sessions of her meditation course and stopped responding to the daily questionnaires in Week 5 of the treatment. However, she had meditated very conscientiously at home for 20–30 min nearly every day. Unfortunately, we do not know whether there were other reasons that caused her to drop out of the study. When we contacted our participants after 12 months, she stated that the course had a very positive impact on her and that meditation proved to be a valuable resource in her life. Participant 3 had stopped meditating soon after the course had ended, whereas Participant 38 used meditation and yoga practice regularly as a means to cope with tension or establish mental calm. The latter stated that the topics of ethical education were often present in her mind.

To allow for better comparison, we also looked at the qualitative statements of the three participants with the highest positive effect sizes. For Participants 14 and 15, the participation in the course (both ME) had led to profound changes in perspective. Specifically, the ethical education component had informed their actions and thoughts in their daily lives up to 12 months after the study had ended. They both continued to meditate: Participant 14 meditated daily and Participant 15 once or twice a week. The former also enthusiastically described how the course had inspired her to follow a spiritual path and form a group of like-minded people to regularly meditate and exchange. Participant 22 (MY) described how she had dived into an intensive yoga practice after the course, which she continued up to the present. She did not continue to meditate. Interestingly, Participants 14 and 15 found meditating very easy from the very beginning of the treatment. All three participants reported meditation becoming increasingly easy over the course of time.

**Figure 4:**
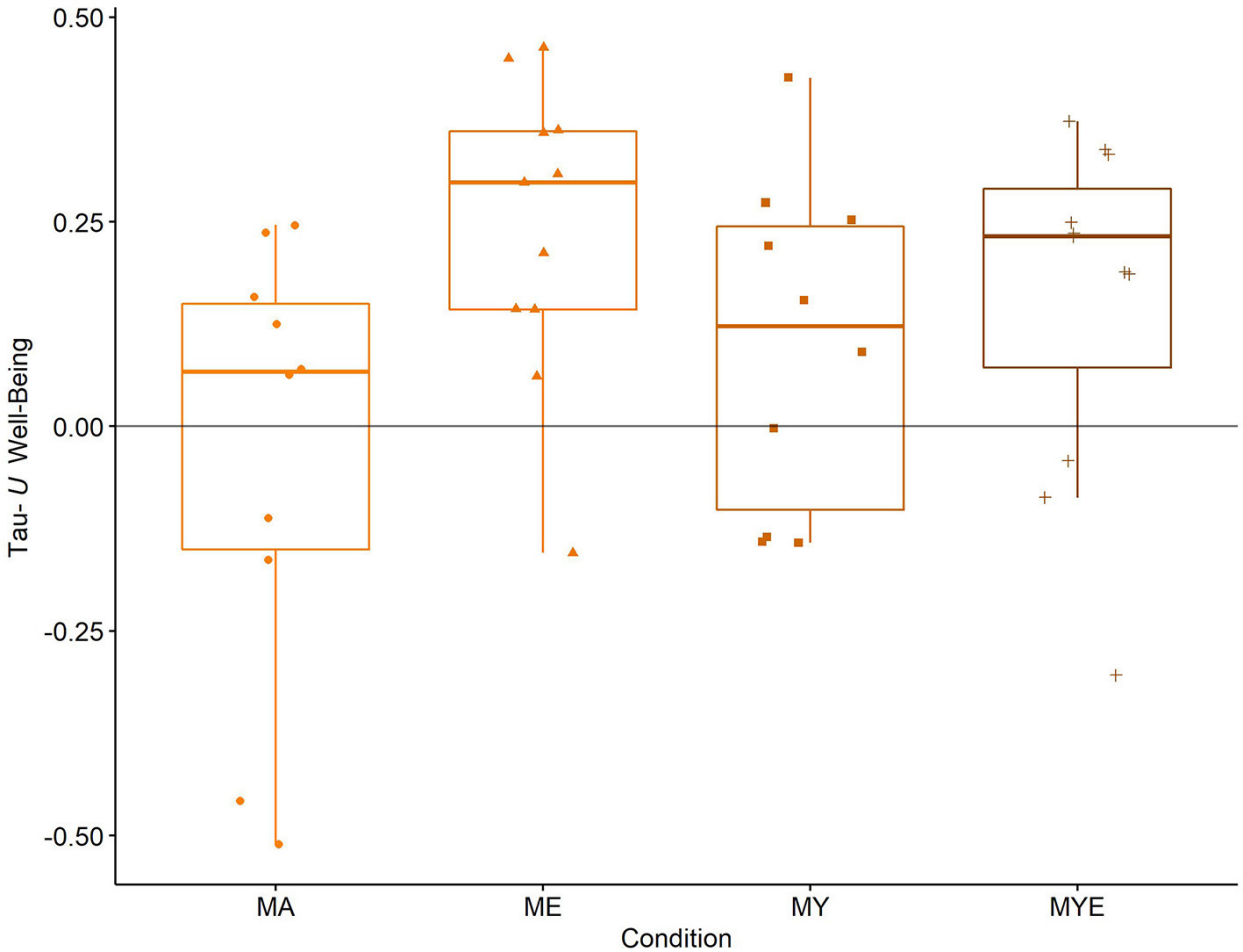
Box plots for averaged Tau-*U* well-being estimates in each condition. Individual well-being estimates are scattered across the box plots. MA, Mantra meditation only; ME, meditation and ethical education; MY, meditation and physical yoga; MYE, meditation, physical yoga, and ethical education. Whiskers represent the largest and lowest values within a distance of 1.5 times the interquartile range.

After this qualitative evaluation, we grouped all Tau-*U* effect size estimates by condition and generated according box plots (Figure 4).

The box plots in Figure 4 reinforce our impression from visual inspection. On average, the MA condition had no effect on participants' well-being [*Mdn* = 0.07, interquartile range (IQR) = 0.30]. All other conditions, however, enhanced participants' well-being. This was particularly pronounced in the two conditions involving ethical education, ME (*Mdn* = 0.30, IQR = 0.22) and MYE (*Mdn* = 0.23, IQR = 0.22), and less pronounced in the MY condition (*Mdn* = 0.12, IQR = 0.35).

We further statistically explored these differences using multiple regression analysis. We entered the effect size estimates as the dependent variable and the abovementioned dummy and control variables as predictors (see Supplementary Material B—Table B4 for a correlation matrix of all variables). The aggregated effect of all four conditions was *F*_(3,33)_ = 2.78, *p* = 0.028, pointing to existing differences between them. The condition model indicated a significant effect for the ME condition, β = 0.56, *p* = 0.006, the MYE condition, β = 0.51, *p* = 0.028, and, pointed to an effect of the MY condition, β = 0.30, *p* = 0.066 (see Supplementary Material B—Table B5 for the full regression table). The component model provided evidence for the effectiveness of the ethical education, but not the physical yoga component in improving subjective well-being (Table 4).

**Table 4:** Regression model for Tau-*U* well-being estimates as dependent variable and effective component, age, gender, and baseline length as predictors (*df* = 36).

**Variable**	***b***	**β**	***SE***	***t***	***p***
(Intercept)	0.32	0.00	0.21	1.55	0.131
Ethical education (yes/no)	0.20	0.44	0.09	2.33	0.013
Physical yoga (yes/no)	0.08	0.17	0.09	0.93	0.179
Total practice time	0.00	−0.28	0.00	−1.36	0.183
Age	−0.01	−0.24	0.01	−1.12	0.270
Gender	0.03	0.06	0.10	0.36	0.723
Occupation	0.05	0.07	0.14	0.32	0.755
Baseline length	0.00	−0.08	0.01	−0.48	0.635

Neither practice time, age, gender, occupation nor baseline length significantly predicted changes in well-being over time in any model. The multiple *R*^2^ was 0.22, indicating that there was still unexplained variance in this model. In summary, it seems that the ethical education component had a positive effect on well-being whereas MA or in combination with physical yoga did not. Conversely, yoga seemed to buffer the negative effect of meditation alone.

##### Multilevel Modeling

We used a similar procedure for multilevel modeling. Time slopes were modeled as random effects. When we estimated the effect of all dummy variables taken together, that is, the effect of the group factor, we found a significant effect of time, *F*_(1,2,492)_ = 6.73, *p* = 0.005, and a significant cross-level interaction between time and condition, *F*_(3,2,492)_ = 3.53, *p* = 0.007. The significant effect of time indicates that well-being did not change during the baseline phase, but gradually increased over the course of the treatment for the majority of participants irrespective of their condition. The significant interaction, on the other hand, suggests differential improvements, such as some conditions yielding stronger improvements than others. In the condition model, there was a significant effect of time, β = 0.08, *SE* = 0.03, *p* = 0.004, indicating a global improvement across all treatments. However, all three interaction terms were significant, too, ME: β = 0.13, *SE* = 0.04, *p* = 0.001, MY: β = 0.09, *SE* = 0.04, *p* = 0.011, MYE: β = 0.09, *SE* = 0.04, *p* = 0.011 (see Supplementary Material—Table B6). This speaks to the supplementary benefit of all three combined interventions and suggests that well-being scores in the combined conditions, particularly in the ME condition, showed a steeper upward slope in the treatment phase. The component model is depicted in Table 5.

**Table 5:** Multilevel regression estimates for well-being scores as dependent variable and time, effective component, age, gender, and baseline length as predictors.

**Variable**	**β**	***SE***	***df***	***t***	***p***
Time	0.08	0.03	2493	2.45	0.007
Ethical education (yes/no)	−0.01	0.12	34	−0.11	0.912
Physical yoga (yes/no)	−0.07	0.12	34	−0.61	0.547
Total practice time	0.15	0.12	34	1.24	0.222
Age	0.07	0.12	34	0.53	0.600
Gender	0.08	0.10	34	0.84	0.407
Occupation	−0.17	0.13	34	−1.31	0.198
Baseline length	0.00	0.10	34	0.01	0.993
Time * Ethical education	0.07	0.03	2493	2.09	0.018
Time * Physical yoga	0.03	0.03	2493	0.92	0.178

There was a significant effect of time as well as a significant interaction between time and ethical education. None of the moderators was a significant predictor of well-being. Thus, it seems that the treatment had an overall positive effect on participants' well-being, but conditions involving ethical education produced stronger enhancements than those that did not. Marginal *R*^2^ of this model was 0.05, and conditional *R*^2^ was 0.45, indicating that only 5% of the variance could be explained by the fixed effects time, components, practice time, age, gender, occupation, and baseline length, whereas 45% was attributable to individual differences.

All three analyses converge, suggesting a generally positive effect of all four treatments on our participants' well-being. Additionally, all analyses show that the combined interventions were more effective than the simple meditation intervention, and that the ethical education component was particularly beneficial in this regard. This effect was independent of the accumulated amount/length of all home practices participants completed. We explore possible explanatory and moderator variables for these findings at the end of the Results section.

### Stress

#### Visual Analysis

**Figure 5:**
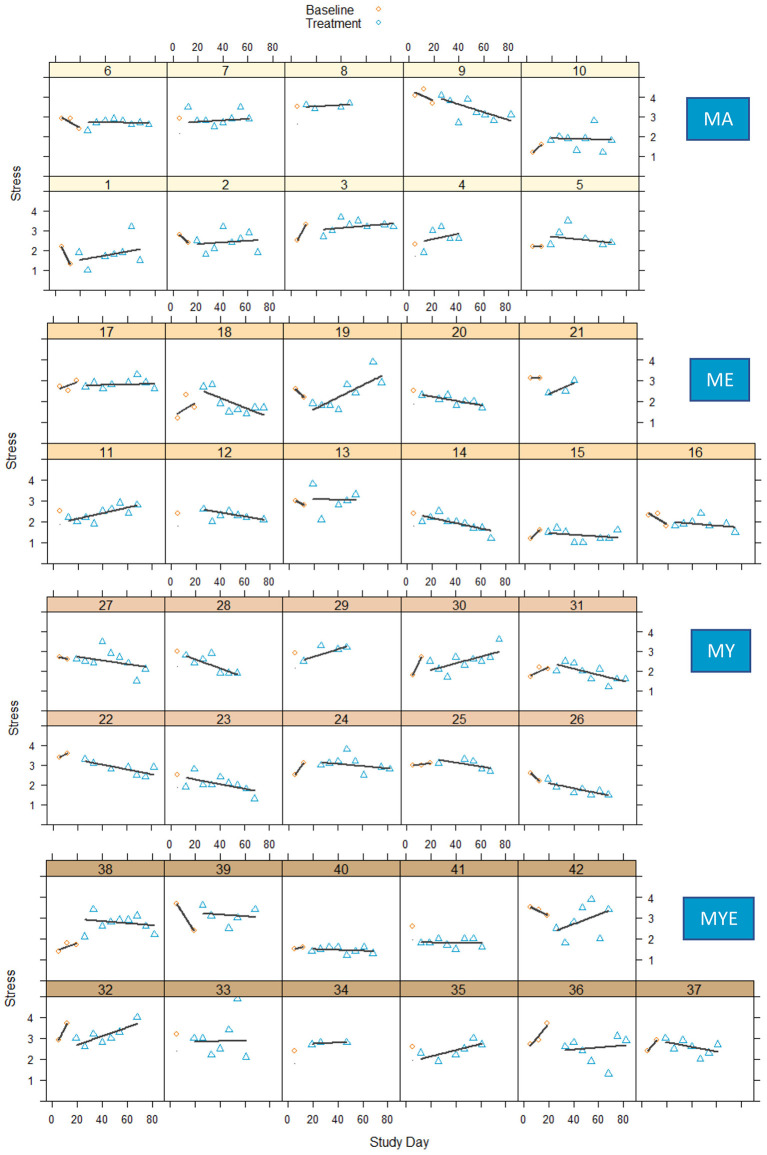
Stress scores during baseline and treatment phases for each participant with regression lines for each phase. MA, Mantra meditation only; ME, meditation and ethical education; MY, meditation and physical yoga; MYE, meditation, physical yoga, and ethical education.

Figure 5 displays weekly stress scores for each participant over the course of the study.

Compared to well-being, perceived stress seemed to fluctuate a lot less. Again, there was substantial variation in participants' general stress levels, their weekly fluctuations as well as their response to the treatment. It was hard to make reliable inferences on baseline trends as there were too few data points in the baseline phase. Cautiously comparing both phases, most treatment curves show a slight to considerable downward trend, indicating reduced stress levels. Only a few participants exhibited unchanging stress levels from baseline to treatment phase (e.g., Participants 7, 33, 39). However, corresponding to our analyses of well-being, some participants experienced an increase in perceived stress. This might possibly have been due to the heightened effort required to participate in our indeed quite demanding study. It seems that the treatment was particularly demanding for participants in the most extensive condition MYE as well as for participants in the MA condition. Conversely, most participants in the MY condition exhibited a consistent reduction in perceived stress. This was also true for a large proportion of participants in the ME condition.

#### Statistical Analysis

##### Tau-U

We had to rely on far fewer measurements for this calculation of effect sizes. Particularly estimates for participants with a baseline length of 7 days need to be interpreted with care. On average, effect size estimates are markedly larger than effect size estimates of well-being and range from −1.00 to 0.87. Yet, most effect sizes range from −0.74 to 0.40, indicating moderate to very large effects on perceived stress. A reduction of perceived stress was desirable—thus, a negative sign in effect sizes represented a change in the expected direction. Whereas the stress level increased for 16 participants, it decreased for 22 participants. Only three participants had an effect size close to zero. The full Tau-*U* table can be found in Supplementary Material B (Table B7).

**Figure 6:**
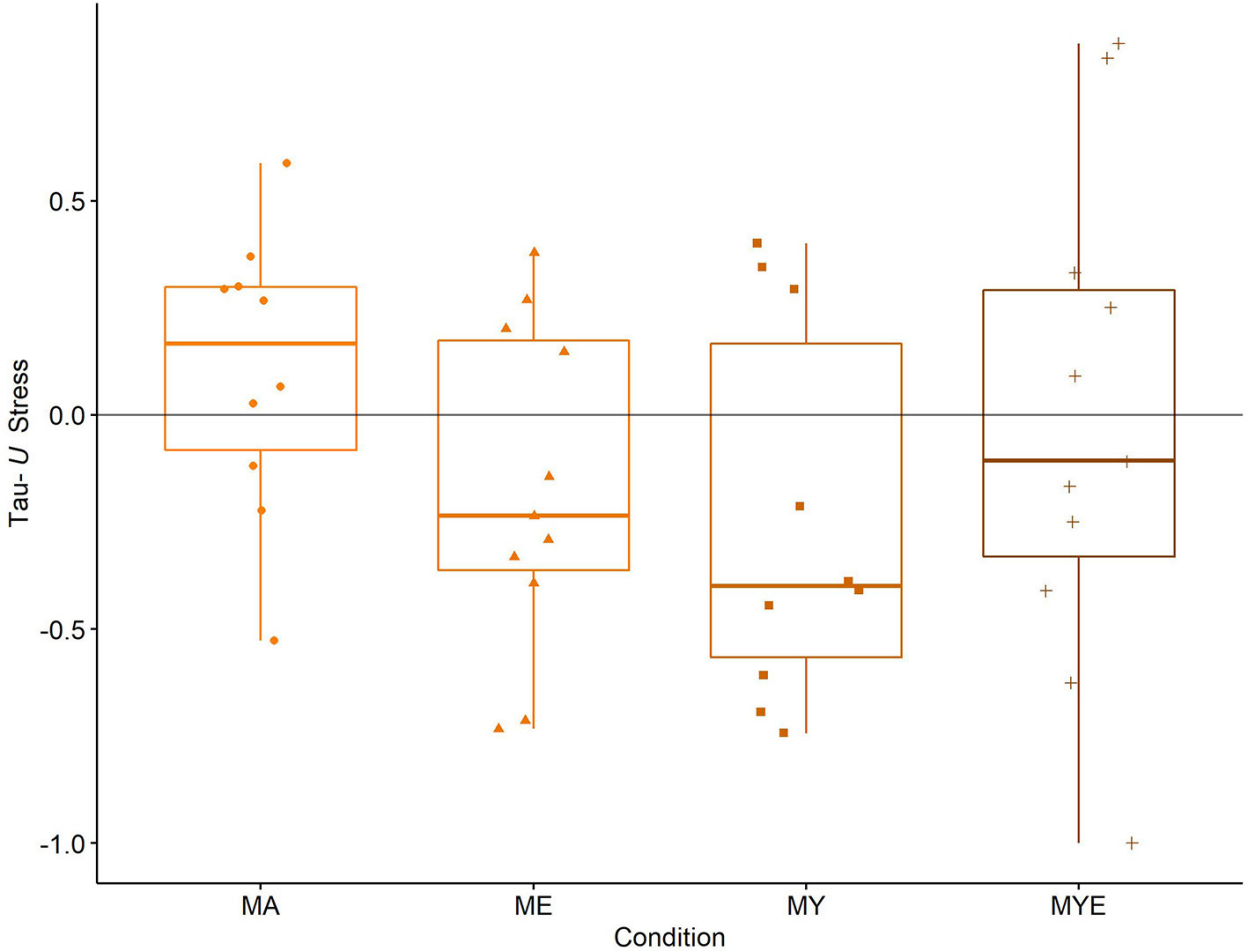
Box Plots for averaged Tau-*U* stress estimates in each condition. Individual stress estimates are scattered across the box plots. MA, Mantra meditation only; ME, meditation and ethical education; MY, meditation and physical yoga; MYE, meditation, physical yoga, and ethical education. Whiskers represent the largest and lowest values within a distance of 1.5 times the interquartile range.

Six participants exhibited large decreases in perceived stress over time, and Participant 41 showed a very large decrease. Three of these received the MY treatment. Indeed, the majority of participants in the MY condition reported decreased levels of stress throughout the treatment, in contrast to most other conditions. Participants 34 and 38 (both MYE condition) showed very large increases in perceived stress over time. The latter had already been identified as experiencing significant decreases in well-being (see above). This was not true for Participant 34, but, she had only very few measurement points in total, indicating an overestimation of the effect. Again, it seems as if the MA condition had the least favorable effect, as half of the participants in this condition exhibited moderate increases and only two moderate decreases in stress. We further explored these apparent differences between conditions by generating box plots for each condition (Figure 6).

The box plots in Figure 6 underpin our impression from the visual inspection and qualitative evaluation. On average, meditation alone (MA) slightly increased perceived stress (*Mdn* = 0.17, IQR = 0.38). In contrast, the treatment helped reduce stress a little in the MYE condition (*Mdn* = −0.11, IQR = 0.62) and to a moderate amount in the ME condition (*Mdn* = −0.24, IQR = 0.54) and the MY condition (*Mdn* = −0.40, IQR = 0.73).

Furthermore, we performed the same regression analyses as for well-being. When we estimated the total effect, we found no differences between conditions, *F*_(3, 33)_ = 1.20, *p* = 0.161. This was true also for the condition model (see Supplementary Material B—Table B8) and the component model (Table 6). Neither practice time, age, gender, occupation nor baseline length significantly predicted perceived stress in either model. Multiple *R*^2^ of the component model was 0.07.

**Table 6:** Regression model for Tau-*U* stress estimates as dependent variable and effective component, age, gender, and baseline length as predictors (*df* = 36).

**Variable**	***b***	**β**	***SE***	***t***	***p***
(Intercept)	−0.12	0.00	0.44	−0.27	0.786
Ethical education (yes/no)	0.03	0.04	0.18	0.18	0.430
Physical yoga (yes/no)	−0.02	−0.02	0.18	−0.12	0.452
Total practice time	0.00	−0.15	0.00	−0.66	0.512
Age	0.01	0.10	0.01	0.44	0.664
Gender	0.21	0.18	0.20	1.03	0.310
Occupation	−0.02	−0.02	0.30	−0.07	0.947
Baseline length	0.00	−0.01	0.01	−0.04	0.972

Apparently, the trends we observed during visual analysis were not as substantial. Nevertheless, the slightly negative effect of the MA condition corresponds to the results we found for well-being. As we expected, Tau-*U* estimates of well-being and stress correlated considerably (*r* = −0.41).

##### Multilevel Modeling

Time was modeled as a random slope. Results differed from those we found with effect size estimates. Overall, there was no significant effect of time, *F*_(3, 331)_ = 1.69, *p* = 0.097, nor a significant cross-level interaction, *F*_(3, 331)_ = 0.97, *p* = 0.205. This also applied to the component model (see Table 7).

**Table 7:** Multilevel regression estimates for stress scores as dependent variable and time, effective component, age, gender, and baseline length as predictors.

**Variable**	**β**	***SE***	***df***	***t***	***p***
Time	−0.06	0.05	332	−1.19	0.117
Ethical education (yes/no)	0.02	0.14	34	0.13	0.900
Physical yoga (yes/no)	0.30	0.14	34	2.09	0.044
Total practice time	−0.37	0.15	34	−2.45	0.020
Age	−0.19	0.15	34	−1.24	0.224
Gender	−0.09	0.12	34	−0.76	0.453
Occupation	0.29	0.16	34	1.82	0.078
Baseline length	0.07	0.12	34	0.59	0.556
Time * Ethical education	0.01	0.05	332	0.13	0.447
Time * Physical yoga	−0.04	0.05	332	−0.82	0.207

Interestingly, accumulated practice time was a significant predictor in this model, indicating that the more participants engaged in a regular home practice the more their stress decreased, independent of the condition they were assigned to. Obviously, total practice time was longer in more extensive conditions, but still varied across participants.

In the condition model (see Supplementary Material B—Table B9), the cross-level interaction between time and the MY condition was significant, β = −0.09, *SE* = 0.06, *p* = 0.051, suggesting that this condition led to the greatest reductions in perceived stress. The magnitude of change was comparable to results obtained for well-being. Yet, two variables had an even greater impact on perceived stress. Practice time was, again, a significant predictor of stress reduction, β = −0.42, *SE* = 0.14, *p* = 0.006, whereas being employed (compared to being a student) significantly predicted an increase of stress, β = 0.37, *SE* = 0.15, *p* = 0.023. In this model, marginal *R*^2^ was 0.22 and conditional *R*^2^ was 0.64.

In sum, findings on stress are inconclusive. Whereas the visual analysis of the line graphs and box plots indicated a stress-relieving effect of all combined treatments, but especially the MY condition, both types of regression analyses did not uncover a significant effect of time or meaningful differences between conditions. Contrary to well-being, engagement in home practice significantly predicted stress reduction in multilevel modeling. It seems that the total amount of home practice was more relevant for a successful stress reduction than the actual treatment participants completed.

### Life Satisfaction

A mixed two-way ANOVA yielded a significant effect of time, *F*_(2, 72)_ = 3.46, *p* = 0.037, with a small effect size, η^2^ = 0.01. The four groups did not differ in their overall life satisfaction, *F*_(3, 36)_ = 1.27, *p* = 0.301; nor was there a significant interaction between time and group, *F*_(6, 72)_ = 0.51, *p* = 0.796. The mean life satisfaction across all groups was *M*_pre_ = 4.99 (*SD* = 1.16) before the study, *M*_post_ = 5.24 (*SD* = 1.12) at completion of the study, and *M*_fu_ = 5.24 (*SD* = 1.14) 2 months later. Thus, life satisfaction increased from pre- to posttest for all participants from our study, and this increase remained stable until follow-up. This increase did not depend on the specific treatment they received. Accordingly, learning how to meditate seems to be sufficient to experience increased life satisfaction.

### Potential Explanatory Variables

We now explore a few variables that might help explain some of the inter-individual variance we observed in the main analyses. For this analysis, we employed mainly exploratory and descriptive methods, such as figures, correlations, and frequency tables.

#### Course Satisfaction

Apart from one participant (Case 4) in condition MY who attended class only once and primarily practiced on her own, class ratings were high in all conditions (the maximum rating being 4)—MA: *Mdn* = 3.44; ME: *Mdn* = 3.75; MY: *Mdn* = 3.38; MYE: *Mdn* = 3.62. Participants in the two conditions that involved ethical education reported a somewhat higher satisfaction with their course, *F*_(1,39)_ = 3.99, *p* = 0.053, which might explain the higher course adherence in these two groups.

#### Adverse or Extraordinary Events

We required participants to mark all symptoms they had experienced throughout the study that were directly related to their practice. These symptoms could be positive (strong positive emotions during meditation), negative (fear, emotional distress), or neutral (feeling hot or cold). Unfortunately, we did not assess whether participants rated their experiences as adverse or not. We transformed duration ratings numerically to resemble comparable intervals. For each cluster of symptoms, we summed up number, severity, and duration ratings to calculate cluster scores. Two participants reported no symptoms, but this could as well represent a lack of diligence. Therefore, we excluded them from the following analyses.

All symptoms were mentioned by at least one participant. The five most common symptoms were exhaustion (54%), impression that something is missing in life (41%), inner tension (39%), strong positive feelings during meditation (33%), and the feeling of oneness with all that is (33%). The six least common symptoms, mentioned by only one participant each, were fainting, redness of the skin, sweating, losing interest in one's surroundings, impression that not meditating is a waste of time, and impression that only people who meditate are valuable people. The most affecting symptoms in terms of number, severity, and duration were reported in the cluster of somatic symptoms (mean affecting score *M* = 7.98), followed by emotional symptoms (*M* = 5.92), and altered states of consciousness (*M* = 5.33). On average, participants reported experiencing 11.8 symptoms (*SD* = 9.57, range 1–44) with a mild severity (*M* = 1.42, *SD* = 0.35) for around 6.2 days (*SD* = 4.26). No adverse events necessitated referral to a health professional.

Participants in the MYE condition reported the most symptoms (altogether 138), followed by those in the MA condition (127) and the ME condition (117). The fewest symptoms were reported by the MY group (77). We tested all symptom clusters for significant differences between conditions employing one-way ANOVAs. Thereby, we found potentially meaningful differences in the number and severity of emotional symptoms, *F*_(3, 35)_ = 2.48, *p* = 0.077, and *F*_(3, 35)_ = 2.81, *p* = 0.054, respectively, and the duration of neurological symptoms, *F*_(3, 35)_ = 2.77, *p* = 0.056. Pairwise *t*-tests revealed that participants in the MY and ME conditions experienced significantly fewer emotional symptoms than those in the MA condition, and, furthermore, participants in the MY condition experienced them as less severe. On the other hand, the ME group experienced significantly longer neurological symptoms than the other groups, but, specifically the MY group. A detailed analysis revealed that these neurological symptoms were predominantly a numbness of body regions, a shaking of the body, and involuntary body movements. It seems, thus, that practicing physical yoga prevents some of the possibly adverse symptoms associated with the practice of mantra meditation and ethical education.

#### Daily Practice

Throughout the treatment, we daily asked participants to rate their experiences with their respective home practice/s. We now present a qualitative review of the changes and differences between conditions that we observed during visual inspection (see Supplementary Material C—Figures C4–C11). For all variables, we observed substantial variation between participants as well as strong day-to-day fluctuations within participants.

For perceived *meditation ease*, we detected a clear upward trend over time, indicating that for most participants meditation got easier over the course of the intervention. This trend was most evident in participants in the ME condition. For some participants, meditation ease stayed more or less the same over time. These participants mainly belonged to the MA and MY conditions. Three participants in the MYE condition experienced increased difficulty in meditating toward the end of the treatment. The visual analysis of perceived *relaxation during meditation* revealed a transition from restlessness to a more relaxed state over time for the majority of participants. Still, ratings of perceived relaxation varied strongly from day to day, suggesting that meditation quality strongly depended on mood and daily form. There were no apparent differences between conditions. *Wakefulness during meditation* showed a similar pattern. The majority of participants experienced a shift from being tired during meditation to being more wakeful, particularly participants from the MY condition.

For *yoga experience* variables, there were no perceivable differences between the two conditions that received physical yoga as a treatment. Wakefulness and relaxation during yoga exercises were consistently high to very high for most participants. In contrast, perceived ease of yoga exercises and their coherence with the breath increased over time for most participants. There were no consistent findings regarding perceived *ease of ethical exercises*. This might have been due to the heterogeneity of weekly topics participants were supposed to engage with. During our weekly meetings, participants repeatedly reported that some topics were more challenging for them than others. To test this assumption, we conducted a simple regression analysis with ease of ethical practice as dependent variable and topic of ethical education as predictor. We set the hardest topic as the reference category (truthfulness) and found that only two topics were significant predictors of perceived ease—non-stealing, *b* = 0.50, *SE* = 0.14, *p* < 0.001, and contentment, *b* = 0.62, *SE* = 0.16, *p* < 0.001. Furthermore, we found substantial correlations between the subjective experience variables, indicating that on “good” days participants perceived all of their home exercises as easier and were more relaxed and awake during meditation and/or yoga (for more details see Supplementary Material A—Section A5).

### Dose–Response and Experience–Response Relations

We evaluated the effects of dosage and subjective experience on the daily fluctuations in our dependent variables during the treatment phase by adding four predictor variables on Level 1 (daily meditation practice duration, perceived meditation ease, relaxation, and wakefulness) to the component models described above. Similar to in other studies (Fredrickson et al., 2017), we used unstandardized values in our models and person-mean centered the meditation variables. In the following, we point out main findings; for full multilevel regression tables see Supplementary Material B (Tables B10, B11). All of these models take into account only measurement points from the treatment phase. Thus, they do not allow for comparisons between baseline and treatment phases.

For well-being, we found significant positive effects for all subjective meditation experience variables (all *p* < 0.02 to *p* < 0.001), but not for meditation practice duration. This means that participants who experienced meditation as easier and were more relaxed and awake during meditation on a given day, compared to their own typical level of daily experience, reported higher levels of well-being on that day. Surprisingly, this effect was independent of the duration of their meditation practice. Unfortunately, we cannot tell from our data whether participants experienced higher well-being because of their meditation, or whether their meditation was easy because they were feeling well. For stress, only relaxation during meditation predicted lower stress levels on a given day (*p* < 0.05).

We were able to explore the direction of these effects tentatively by considering the time of meditation practice that participants reported. We found that meditating in the morning positively influenced well-being on that day and that participants meditated less on days when they were feeling well and more when they were feeling less stressed (for more details see Supplementary Material A—Section A6).

## Discussion

The present study provides the first in-depth insights into the incremental impact of ethical education and physical Hatha yoga on mantra meditation in healthy participants. At the same time, it dismantled and investigated diverse combinations of the components of the new MBLM mind–body therapy (Bringmann et al., 2020), which is based on the yoga path. The single-case multiple-baseline design gave detailed access to individual responses and trajectories of change. Participants in all four conditions enjoyed their course and established a regular home practice, indicating that MBLM is a feasible and helpful intervention for a predominantly young and healthy population. Course satisfaction and adherence was a bit higher in conditions that involved ethical education. Ethical education also had the greatest impact on increasing participants' well-being. While results on well-being were quite strong and unambiguous, findings on stress were inconclusive. Overall, the majority of participants experienced an increase in well-being and a decrease in stress over time. However, for both variables, the combined interventions had more positive effects on participants than the simple meditation intervention.

For stress, changes could not be consistently attributed to the inclusion of a specific component. The MY condition was the most efficient in reducing stress, tentatively speaking to a stress-relieving effect of physical yoga. The MA condition was the least effective as some participants showed a decrease in well-being and/or an increase in stress over time. The positive effects of ethical education on well-being were independent of the total amount of home practice participants completed, suggesting a benefit specific to this yoga component. These results provide evidence for the differential effects various combinations of yoga components can elicit.

Interestingly, life satisfaction significantly improved across all conditions from pre- to posttest and had continued to improve when measured at 2 month follow-up. Thus, participating in any of our four interventions, whether simple or complex, seemed to be beneficial for contentment in life. This might be an effect specific to mantra meditation, as all conditions involved this practice. Alternatively, it might be due to unspecific factors common to all conditions, such as group dynamics, social support, or attention from study staff. Yet, the latter might be unlikely as the effect persisted until follow-up. From a eudaemonic perspective, decreased hedonic well-being, as reported above for the MA condition, is not inconsistent with increased life satisfaction (Ryan and Deci, 2001). Even if participants felt more stressed during the treatment, they might have gained profound insights during meditation that significantly affected their perspective and satisfaction with life.

Overall, we observed high inter- as well as intrapersonal variability in responses emphasizing the potential relevance of personality factors in this regard. While some participants benefited strongly from their treatment, others did not change much, and still others experienced a deterioration in their well-being or an increase in perceived stress. Similarly, for some participants meditating was really easy from the beginning, for most it got easier as they practiced, and for some it remained difficult throughout. The different dimensions of subjective experience during home practice were interrelated, suggesting that meditation or yoga exercises were easier when participants felt relaxed and awake. Physical yoga might be helpful in this regard as participants in the MY condition experienced the most prominent increase in wakefulness during meditation. Ethical education, on the other hand, can be quite challenging or unsettling and thus can impair relaxation during meditation. From our observations of the different classes, the transition to meditation was much smoother and quieter in the conditions where meditation was preceded by physical Hatha yoga (MY and MYE) than in the ME condition. Nonetheless, the latter showed the greatest improvement in perceived meditation ease. As this condition also showed the greatest increases in well-being, it appears to have been a very effective combination. The combination of physical yoga and meditation (MY), though, seems to have been particularly beneficial in reducing stress and adverse events associated with the treatment.

### Framing Mantra Meditation Enhances Its Effects

It is not easy to compare our findings to results of earlier studies. Although there have been some comparative or dismantling studies (Matko et al., 2021a), no study employed an additive design comparable to ours. Most studies compared rather complex interventions with each other, and only a few actually dismantled or added program components (e.g., Smith et al., 2011; Hunt et al., 2018). Only one study compared a complex Kundalini Yoga program (including meditation, breathing, and some movement) to a meditation program entailing mantra and breathing meditation (Shannahoff-Khalsa et al., 2019) and found that the complex yoga program^1^ outperformed the simple meditation program. This is in line with the results of our study. Conversely, it is not quite clear why the mantra meditation condition in our study elicited no changes or even negative effects. Mantra meditation has been shown to have a strong impact on negative emotions, stress, anxiety, and depression, but not necessarily well-being (Sedlmeier et al., 2012; Lynch et al., 2018). However, there are to date only a few investigations into the effects of mantra meditation on healthy participants that are methodologically sound and more research into this matter is needed.

Interestingly, our mantra meditation intervention differed significantly from earlier investigations. The format of teaching mantra meditation might not have been optimal. Participants received only minimal instructions and were then “thrown in at the deep end” with instructions to immediately begin practicing 20–25 min of silent meditation. Research has shown that letting participants engage in a guided meditation practice resulted in greater improvements than letting them engage in silent meditation (Trivedi et al., 2020). Mantra meditation has been proposed as a suitable practice for both beginners and advanced meditators (Devananda, 1999), and also for patients with mental disorders (Orme-Johnson and Barnes, 2013). Nevertheless, individual factors might influence the liking of and coping with a specific meditation technique and, therefore, its effects (Hölzel et al., 2011). We assessed a multitude of personality factors in the larger project this study belongs to, and will explore possible interactions in future publications. Another factor could have been that participants were fully informed about the experimental procedure and might have been disappointed at receiving only the minimal treatment. Accordingly, comparing equally extensive interventions, for example, diverse meditation techniques, might lead to different effects.

Furthermore, interventions in other mantra meditation studies were embedded in a spiritual framework and enriched by rituals, additional exercises, or a sense of secrecy or sacredness. Most mantra meditation programs follow a specific spiritual teacher or lineage (Kirtan Kriya, Mantram Repetition, Passage Meditation, and Transcendental Meditation). Research has shown that spirituality is a critical ingredient in mantra meditation and can tremendously enhance its effects (Wolf and Abell, 2003; Wachholtz and Pargament, 2005). Although we employed spiritual mantras in this study, we did not provide any additional information on the belief systems or spiritual entities behind these mantras. Thus, our mantra meditation intervention was rather technical and less devotional than other programs. This might have impaired its effectiveness.

Indeed, providing participants with some kind of framework, such as physical yoga or ethical education, reversed the negative effect of mantra meditation in this study. While ethical education provided a philosophical framework to contextualize the practice of mantra meditation as well as experiences made during meditation, physical yoga offered a bodily or embodied framework. Yoga postures and breath work help people calm body and mind and develop a better connection to and understanding of their own bodily processes (Schmalzl et al., 2015; Kishida et al., 2018). Traditionally, postures and breathing were considered preparatory exercises that preceded meditation and helped the yogi reach the “stilling of the changing states of the mind” described in the Yoga Sutras (Bryant, 2015). Likewise, the ethical practice of the *yamas* and *niyamas* was supposed to ground and permeate all other yogic practices, such as postures or meditation (Feuerstein, 2012).

Following these assumptions, the full MBLM program (MYE) should have led to the greatest effects, but this was not the case. It seems, rather, that certain combinations of practices were more helpful than others and effects could not be reduced to simple dosing effects. Indeed, in this sample of healthy adults, a bit more was better than much more as the ME and MY treatments outperformed the MYE treatment. One reason for this finding might be the substantially longer class duration in the MYE condition as well as the larger amount of assigned home practice. Interestingly, in a systematic review, effect sizes did not change in shortened vs. original MBSR treatments, and shorter assigned practice time was associated with larger effect sizes (Carmody and Baer, 2009). In our study, the total amount of home practice participants completed was not related to well-being outcomes, but was related to stress outcomes. This indicates that less extensive interventions are probably easier to integrate into people's lives, but more practice helps to reduce stress more. Furthermore, the eight-fold yoga path was designed as a lifelong journey for spiritual seekers on their way to enlightenment (Feuerstein, 2012). Participants in scientific studies (and meditators in general) are usually motivated to meditate for much more mundane reasons (Sedlmeier and Theumer, 2020). Thus, providing participants with a less extensive set of practices might give them more time to adjust and assimilate.

### Specific Combinations of Practices Yield Different Effects

In this context, which component of the yoga path or, rather, which combination of components is most effective? We cannot give a comprehensive answer to this question, as we investigated only four possible combinations of practices, but will tentatively discuss our findings in the following. The intensive ethical confrontation in the ethical education groups invited participants to reconsider some of their maladaptive cognitive, emotional, and behavioral habits and divert them to a more adaptive direction. Essentially, this is one of the core principles of cognitive-behavioral therapy and stress reduction programs (Lehrer et al., 2007; Powers et al., 2017).

The ethical education component of MBLM might help attendees gain a deeper awareness of their goals and values in life, empowering them to make adaptive choices and switch off the “automatic pilot” of daily actions. Acting in accordance with personal values has positive effects on well-being and quality of life (Brunstein, 1993; Franquesa et al., 2017). Likewise, the importance of value-related behavior has become increasingly popular in psychotherapy, for example, in acceptance and commitment therapy (Hayes et al., 2004), and in positive psychology in general (Seligman, 2004). It has been suggested as a potential mechanism of mindfulness (Kocovski et al., 2009) and yoga (Sullivan et al., 2017) interventions. Our findings are also in line with research demonstrating that incorporating ethical practice into yoga or mindfulness interventions increased their efficacy (Smith et al., 2011; Chen and Jordan, 2020). Remarkably, engagement in intergroup discussions was strongest in the ME condition, even after the class had ended. In contrast to all other conditions, these participants also formed an informal meditation group and continued to meet after they finished the study. It might well be that this group initiated a self-reinforcing process that boosted the treatment's efficacy.

Engaging in physical Hatha yoga practice and simple breathing exercises might have initiated an upsurge in resilience to stress (Hartfiel et al., 2011; Manincor et al., 2016). Perceived stress decreased most in this condition and participants reported the fewest and the mildest emotional and other adverse symptoms during the treatment. This speaks to the protective effects of physical yoga practice. Findings from other studies support the positive effect of (physical) yoga on psychological well-being and stress (Bhat et al., 2012; Gard et al., 2012; Gorvine et al., 2019). Conversely, some studies found no effect on stress (Quach et al., 2016; Park et al., 2020). Admittedly, the yoga interventions under investigation in these studies varied greatly, making it hard to draw reliable conclusions. Yet, many of the abovementioned studies used psychological as well as physiological measures of stress. Thus, a multimodal assessment and use of more standardized intervention protocols (Sherman, 2012) in future studies might provide more support for the stress-relieving effect of physical yoga.

Possibly, physical yoga might have enhanced non-judgmental metacognitive monitoring, as the yoga instructor repeatedly encouraged participants to observe their bodily sensations and thoughts in an accepting and non-judgmental manner. This process has been proposed as a central mechanism in yoga-based practices (Schmalzl et al., 2015) and mindfulness meditation (Lindsay et al., 2018). This accepting stance might have broadened to include the meditation practice, making it easier for participants to meditate. Moreover, physical yoga and breath work have been found to decrease sympathetic response and increase vagal/parasympathetic activity (Gard et al., 2014; Riley and Park, 2015), thereby intensifying the calming effect of yoga. In contrast to participants in the other conditions, participants in the MY condition were the least talkative during and after class and developed the least group cohesion. Our impression was that the MY condition provided participants with a valid opportunity to increase self-care but did not lead to the profound reorientation and reconsideration of values that we observed in the two ethical education conditions.

### Limitations and Future Directions

The single-case multiple-baseline design enabled us to monitor changes in well-being, stress, and subjective experience continuously and with a high time resolution. However, a few features of the study might limit the generalizability of its outcomes. First, we recruited a convenience sample of young healthy participants from the general public, which consisted mainly of students. The majority of participants were 18–36 years old, with two exceptions—two women who were 57 and 61 years old. Nonetheless, age did not significantly predict any of the outcome variables. Still, this sample cannot be considered representative of the general public. Moreover, as participants received no financial compensation for their participation, we have to assume that they were intrinsically motivated to participate in this study and shared an inherent interest in or openness to yoga and meditation. The lack of financial compensation might explain the relatively high number of dropouts and high amounts of missing data and attrition toward the end of the study. Future studies should consider providing financial or other compensation to increase commitment in studies that employ intense data-gathering periods. Furthermore, this approach might also attract individuals who are less intrinsically motivated.

In single-case research, a sample of 42 participants is considered exceptionally large. With up to 85 measurements per participant for daily measures, findings, and effect size estimates should be very robust. This applies less to measures of stress. Usually, single-case research manuals recommend taking at least three to five baseline measurements (Barlow et al., 2009; Kratochwill et al., 2010). Unfortunately, this could not be achieved in all cases in this study. Thus, results on perceived stress should be interpreted with care. Future studies should either increase baseline lengths for all participants or try to capture perceived stress more often. The latter could be achieved by including simple questions in daily assessments, such as “How stressed have you felt throughout the day?”

The sample size was somewhat small for multilevel modeling and pre–post comparisons, though. Multilevel modeling is usually applied to much larger data sets with a large number of data points at both levels. However, simulation studies performed with multiple-baseline data have included fewer participants and measurements occasions and reported satisfactory results (Ferron et al., 2009; Moeyaert et al., 2017). Nevertheless, more research is needed to validate our findings. Likewise, subgroup samples for the four conditions were comparatively small for drawing reliable conclusions through ANOVAs. Nonetheless, we were able to comply successfully with one of the main limitations of yoga and meditation research, that is, finding suitable control groups. The meditation-only condition served as an appropriate baseline group for depicting the effect of meditation as well as factors common to a group setting (Kinser and Robins, 2013; Stein and Witkiewitz, 2020). Intriguingly, our study showed that these factors were maybe not as important and could not outweigh the partially negative effects of mantra meditation.

A couple of improvements could be made to make the treatment and the data collection more feasible and enjoyable for participants. First, it would be advisable to give participants more guidance on mantra meditation. Second, daily questionnaires could be shortened and at the same time be made more specific (see above). Alternatively, experience-sampling methods or ecological momentary assessment (Shiffman et al., 2008) provide an intriguing means to capture immediate experiences at different time points in one day. Furthermore, participants should be encouraged to make journal-like entries in the daily questionnaire and reliably report challenging situations, such as exams. This would enable researchers to understand the fluctuations in their daily experience better. We did provide a field for exceptional experiences in our questionnaire, but not all participants regularly made use of this.

Finally, future studies could dismantle the effects of the eight-fold yoga path in an even more detailed way. Accordingly, studies could compare the effects of combined practices to treatments incorporating only ethics, only physical yoga, in addition to only meditation. Alternatively, they could examine the effects of diverse meditation techniques in this context. Current research has revealed a multiplicity of meditation techniques (Matko and Sedlmeier, 2019; Matko et al., 2021b), most of which are under researched at present. Furthermore, yoga incorporates a collection of diverse breathing techniques that can have quite different, sometimes even opposing effects on practitioners (Raghuraj et al., 1998; Peng et al., 2004). Thus, future studies should examine different combinations of ethical education, postures, breathing practices, and meditation techniques. Dosing questions should be paid special attention in this context. The four conditions we investigated in this study differed in session length, which might have influenced their outcomes. However, the assigned and reported home practice was comparable across the various components and could not account for differences in well-being change. Furthermore, as we mentioned above, the longest treatment was not the most effective. Nevertheless, more studies are needed to truly understand the multifaceted practice of yoga in its entirety. In the end, these research efforts could contribute to the development of a profound theory of yoga.

## Data Availability Statement

All scripts and data that support the results can be found at the Open Science Framework (osf.io/n7y64/).

## Ethics Statement

The studies involving human participants were reviewed and approved by the institutional review board of the Chemnitz University of Technology. The patients/participants provided their written informed consent to participate in this study.

## Author Contributions

KM designed and executed the study, analyzed the data, and wrote the first draft of the manuscript. PS collaborated on the study design and data analysis. HB conceptualized and supervised the interventions and collaborated with the study design and data analysis. All authors worked on the final version of the manuscript.

## Conflict of Interest

The authors declare that the research was conducted in the absence of any commercial or financial relationships that could be construed as a potential conflict of interest.

## Publisher's Note

All claims expressed in this article are solely those of the authors and do not necessarily represent those of their affiliated organizations, or those of the publisher, the editors and the reviewers. Any product that may be evaluated in this article, or claim that may be made by its manufacturer, is not guaranteed or endorsed by the publisher.

## Footnotes

^1^We acknowledge that both groups in the respective study were classified as meditation and our labeling might divert from the traditional Kundalini Yoga (as taught by Yogi Bhajan) view. Yet, as the Kundalini Yoga group also entailed breathing and movement practices (contrary to the Relaxation Response) and, thus, more components from the classical yoga path, we decided to label it a “complex yoga program”.
