# Supplementary material for: Differential Effects of Ethical Education, Physical Hatha Yoga, and Mantra Meditation on Well-Being and Stress in Healthy Participants—An Experimental Single-Case Study
Source: Front Psychol. 2021 Aug 5;12:672301. doi: 10.3389/fpsyg.2021.672301 (PMC8375679; doi:10.3389/fpsyg.2021.672301)
Supplement: Supplementary file 1 [file Data_Sheet_1.pdf]

Supplementary Material A

Study Materials and Further Analyses

Differential Effects of Ethical Education, Physical Hatha Yoga, and Mantra Meditation on Well-Being and Stress in Healthy Participants: An Experimental Single-Case Study

Table A1

Variables Gathered in the Study with the Respective Measurement Instruments and Times

Variable	Instrument	Authors	Measurement Time
**Affectivity**	Affective Grid	Russel, Weiss & Mendelsohn (1989)	Daily
**Daily Practice**	Experiences during meditation, yoga and ethical practices	Own creation	Daily
**Well-Being**	WHO-5 Wellbeing Scale	World Health Organization (1998)	Daily
**Body Awareness**	Adapted Body Awareness Questionnaire	Adapted from MAIA, Mehling et al. (2012); PBCS, Miller et al. (1981); and BAQ, Shields et al. (1989)	Twice weekly
**Mind-Wandering**	Mind-Wandering Questionnaire (MWQ)	Mrazek et al. (2013)	Twice weekly
**Sustained Attention**	Sustained Attention to Response Task (SART)	Robertson et al. (1997)	Twice weekly
**Attention Network**	Attention Network Test (ANT)	Fan et al. (2002)	Weekly
**Decentering**	Experiences Questionnaire Decentering (EQ-D)	Fresco et al. (2007)	Weekly
**Emotion Regulation**	Difficulties in Emotion Regulation Scale (DERS)	Gratz & Roemer (2003)	Weekly
**Motivation**	Situational Motivation Scale (SIMS)	Deci & Ryan (1985)	Weekly
**Self-Reflection of Concrete Changes**	Result-oriented Problem- and Self-Reflection (RoPS-CC)	Greif & Berg (2011)	Weekly
**Stress**	Perceived Stress Scale (PSS-10)	Cohen & Williamson (1983)	Weekly
**Absorption**	Tellegen Absorption Scale (TAS)	Tellegen & Atkinson (1974)	Pretest
**Big Five**	Big Five Inventory (BFI-K)	Rammstedt & John (2007)	Pretest
**Need for Cognition**	Need for Cognition Short Form (NFC-SF)	Cacioppo, Petty, & Kao (1984)	Pretest
**Distress Tolerance**	Distress Tolerance Scale (DTS)	Simons & Gaher (2005)	Pre/Posttest
**Life Satisfaction**	Satisfaction with Life Scale (SWL)	Diener et al. (1985)	Pre/Posttest

**Self-Compassion**	Self-Compassion-Scale (SCS)	Neff (2003)	Pre/Posttest
**Spirituality**	Aspects of Spirituality (ASP)	Büssing, Ostermann, & Matthiessen (2007)	Pre/Posttest
**Trait Anxiety**	State-Trait-Anxiety Inventory (STAI)	Laux et al. (1981)	Pre/Posttest
**Adverse Events**	Adverse Events Questionnaire	Own creation	Posttest
**Course Satisfaction**	Client Satisfaction Questionnaire (CSQ-8)	Attkinsson & Zwick (1982)	Posttest

References

Attkisson, C. C., & Zwick, R. (1982). The Client Satisfaction Questionnaire: Psychometric properties and correlations with service utilization and psychotherapy outcome. *Evaluation and Program Planning*, *5*(3), 233–237.

Büssing, A., Ostermann, T., & Matthiessen, P. F. (2007). Distinct expressions of vital spirituality: The ASP questionnaire as an explorative research tool. *Journal of Religion and Health*, *46*(2), 267–286. JSTOR.

Cacioppo, J. T., Petty, R. E., & Kao, C. F. (1984). The efficient assessment of need for cognition. *Journal of Personality Assessment*, *48*(3), 306–307. https://doi.org/10.1207/s15327752jpa4803_13

Cohen, S., Kamarck, T., & Mermelstein, R. (1983). A global measure of perceived stress. *Journal of Health and Social Behavior*, *24*(4), 385–396. https://doi.org/10.2307/2136404

Deci, E. L., & Ryan, R. M. (1985). The general causality orientations scale: Self-determination in personality. *Journal of Research in Personality*, *19*(2), 109–134.

Diener, E. D., Emmons, R. A., Larsen, R. J., & Griffin, S. (1985). The Satisfaction with Life Scale. *Journal of Personality Assessment*, *49*(1), 71–75.

Fan, J., McCandliss, B. D., Sommer, T., Raz, A., & Posner, M. I. (2002). Testing the efficiency and independence of attentional networks. *Journal of Cognitive Neuroscience*, *14*(3), 340–347.

Fresco, D. M., Moore, M. T., van Dulmen, M. H., Segal, Z. V., Ma, S. H., Teasdale, J. D., & Williams, J. M. G. (2007). Initial psychometric properties of the Experiences Questionnaire: Validation of a self-report measure of decentering. *Behavior Therapy*, *38*, 234–246.

Gratz, K. L., & Roemer, L. (2003). Multidimensional assessment of emotion regulation and dysregulation: Development, factor structure, and initial validation of the Difficulties in Emotion Regulation Scale. *Journal of Psychopathology and Behavioral Assessment*, *26*(1), 41–54.

Greif, S., & Berg, C. (2011). *Result-oriented self-reflection: Report on the construct validation of theory-based scales*. University of Osnabrück. http://www.home.uniosnabrueck.de/sgreif/english/downloads.html

Laux, L., Glanzmann, P., Schaffner, P., & Spielberger, C. D. (1981). *Das State-Trait-Angstinventar*. Hogrefe.

Mehling, W. E., Price, C., Daubenmier, J. J., Acree, M., Bartmess, E., & Stewart, A. (2012). The Multidimensional Assessment of Interoceptive Awareness (MAIA). *PloS One*, *7*(11), Article e48230.

Miller, L. C., Murphy, R., & Buss, A. H. (1981). Consciousness of body: Private and public. *Journal of Personality and Social Psychology*, *41*(2), 397–406. https://doi.org/10.1037/0022-3514.41.2.397

Mrazek, M. D., Phillips, D. T., Franklin, M. S., Broadway, J. M., & Schooler, J. W. (2013). Young and restless: Validation of the Mind-Wandering Questionnaire (MWQ) reveals disruptive impact of mind-wandering for youth. *Frontiers in Psychology*, *4*, Article 560.

Neff, K. D. (2003). The development and validation of a scale to measure self-compassion. *Self and Identity*, *2*(3), 223–250.

Rammstedt, B., & John, O. P. (2007). Measuring personality in one minute or less: A 10-item short version of the Big Five Inventory in English and German. *Journal of Research in Personality*, *41*(1), 203–212.

Robertson, I. H., Manly, T., Andrade, J., Baddeley, B. T., & Yiend, J. (1997). Oops!': Performance correlates of everyday attentional failures in traumatic brain injured and normal subjects. *Neuropsychologia*, *35*(6), 747–758.

Russell, J. A., Weiss, A., & Mendelsohn, G. A. (1989). Affect grid: A single-item scale of pleasure and arousal. *Journal of Personality and Social Psychology*, *57*(3), 493–502.

Shields, S. A., Mallory, M. E., & Simon, A. (1989). The Body Awareness Questionnaire: Reliability and validity. *Journal of Personality Assessment*, *53*(4), 802–815. https://doi.org/10.1207/s15327752jpa5304_16

Simons, J. S., & Gaher, R. M. (2005). The Distress Tolerance Scale: Development and validation of a self-report measure. *Motivation and Emotion*, *29*(2), 83–102. https://doi.org/10.1007/s11031-005-7955-3

Tellegen, A., & Atkinson, G. (1974). Openness to absorbing and self-altering experiences ("absorption"), a trait related to hypnotic susceptibility. *Journal of Abnormal Psychology*, *83*(3), 268–277.

World Health Organization. (1998). Info package: Mastering depression in primary care, version 2.2. *WHO, Regional Office for Europe, Psychiatric Research Unit*. https://www.euro.who.int/__data/assets/pdf_file/0016/130750/E60246.pdf

Section A2

Detailed Description of Each Treatment Component

All four conditions involved learning to meditate using mantra meditation. During a short introduction to the practice, we asked them to choose a mantra from a list of traditional mantras from different spiritual contexts (see Table A3). The key practice in this form of mantra meditation is silently repeating the chosen mantra while letting all other thoughts pass by and letting the breath flow naturally. Participants were encouraged to sit with a straight spine and with their eyes closed. This practice was supposed to help participants develop one-pointed concentration and, gradually, reach a calm and still state of mind. Moreover, participants were encouraged to take a curious and friendly approach toward their experience. Each weekly session in every condition included a 25-min silent (i.e., nonguided) mantra meditation practice. Additionally, we asked all participants to practice mantra meditation at home for at least 20 min each day. They received a comprehensive manual of mantra meditation.

During the physical yoga practice, participants learned a set of simple yoga postures, breathing and relaxation techniques (for more detailed information on yoga practices, see, e.g., Iyengar, 2009; Stephens, 2011). Each yoga class started with approximately 10 min of breathing exercises, followed by 30 min of postures and dynamic exercises, and concluded with a 10-min guided relaxation. The set of yoga exercises was devised prior to the study by KM. She also led all yoga classes during the study, in which the group practiced in synchrony. Yoga classes were designed to advance from simple to more advanced exercises and were adjusted to participants' needs and abilities. Participants received a handout including detailed instructions on each exercise and were asked to engage in physical yoga for at least 20 min every day at home.

Ethical education followed the protocol developed for the MBLM mind–body program (Bringmann et al., 2020). The manualized MBLM training program was extensively pilot tested. A mixed-methods study has indicated that it is a highly acceptable and feasible program for outpatients with mild to moderate depression (Bringmann et al., 2021). The *Ethical Living* domain of MBLM consists of 8 weekly topics based on the *yamas* and *niyamas* of yoga philosophy. Each week, we introduced one of the 10 *yamas* and *niyamas*, with the last three *niyamas* being grouped together for time reasons into one topic called "transcendence." After we introduced each topic, we invited participants to discuss its application and relevance for their daily lives. Participants also received handouts outlining the key aspects of the topic and presenting six related mindful living exercises. We invited them to engage daily in these activities during the following week and write down their experiences. In the next session, they shared and reflected upon their experiences.

<u>References</u>

Bringmann, H. C., Bringmann, N., Jeitler, M., Brunnhuber, S., Michalsen, A., & Sedlmeier, P. (2020). Meditation-Based Lifestyle Modification: Development of an integrative mind-body program for mental health and human flourishing. *Complementary Medicine Research*, 1–11. https://doi.org/10.1159/000512333

Bringmann, H. C., Bringmann, N., Jeitler, M., Brunnhuber, S., Michalsen, A., & Sedlmeier, P. (2021). Meditation Based Lifestyle Modification (MBLM) in outpatients with mild to moderate depression: A mixed-methods feasibility study. *Complementary Therapies in Medicine*, *56*, Article 102598. https://doi.org/10.1016/j.ctim.2020.102598

Iyengar, B. K. S. (2009). *Light on yoga: Yoga Dipika*. Harper Collins.

Stephens, M. (2011). *Teaching yoga: Essential foundations and techniques*. North Atlantic Books.

Table A3

List of Traditional Mantras from Different Spiritual Traditions

Tradition	Mantra
Christian	Mein Gott und mein Alles/ My God and my everything
	Kyrie Eleison
	Jesus/ Lord Jesus Christ
	Ave Maria
Buddhist	Om Mani Padme Hum
	Om Namo Butsaya
Hindu	Om Namah Shivaya
	Om Sri Ram Jay Ram Jay Jay Ram
	Rama
	Om Prema
	Om Shanti
	So Ham
	Am Am Am Atmashakti Avaham
Jewish	Baruch Atah Adonai
	Ribono Shel Olam
	Shalom
Indian	O Wakan Tanka
Bija mantras	Om
(seed syllables)	Hessraim
	Ram
	Ham
	Aim
	Hrim
	Krim
	Klim
	Gam

Table A4

List of Symptom Clusters with Their Corresponding Symptoms

Cluster	Symptoms
Altered states of consciousness	Changed perceptions of time and space
	Feeling of oneness with all that is
	Feelings of strong energy flows/currents during meditation
	Hallucinations/visions/illusions
	Sense of blurring or dissolving of self-world boundaries
	Sense of inner emptiness or vastness
Changes in necessities	Changed appetite
	Changed sexuality
	Changed sleep
Cognitive symptoms	Feeling superior to others
	Impression that only people who meditate are valuable
	Increased awareness of negative features/traits
	Increased criticism of others
	Increased self-criticism
	Mental confusion
	Remembrance of negative experiences
Compulsive meditation	Feeling that the time not spent meditating is wasted
	Need for continuous meditation
	Restlessness/anxiety when not practicing formal meditation
Difficulties in life	Difficulties in participating in everyday life or continuing to work
	Difficulty in feeling comfortable in the world
	Hypersensitivity/rejection of urban life
	Impression that something is missing in life
	Lack of orientation in life
Emotional symptoms	Boredom
	Frightening thoughts
	Inner tension
	Irritability
	Pronounced emotional pain
	Pronounced fear/anxiety
	Strong negative emotions during meditation
	Strong positive emotions during meditation
	Tearfulness

Motivational symptoms	Impression of being bored by others
	Lack of interest in one’s surroundings
	Lack of interest in others
	Lack of interest in people’s conversations
	Less motivation in life
Neurological symptoms	Dizziness
	Drowsiness
	Fainting
	Involuntary body movements
	Involuntary laughting or crying
	Numbness in parts of the body
	Shaking of the body
	Twitching in parts of the body
Pain	Chest pain
	Headache
	Joint pains
	Muscular pains
	Stomachache
Somatic symptoms	Blocked nose
	Coughing
	Diarrhea
	Exhaustion
	Experiencing strong chills or flushing
	Feeling hot or cold
	Feeling unwell/ill
	Heavy head
	Itching
	Itching in the eye
	Muscle cramps
	Nausea
	Palpitations
	Pronounced sweating
	Redness of skin
	Running nose
	Salivation
	Shortness of breath
	Tinnitus

Section A5

Correlations Between Subjective Experience Variables

Next, we looked at whether there were any significant correlations between the subjective experience variables. Perceived meditation ease correlated highly with relaxation during meditation (r = .56) and moderately with relaxation during physical yoga (r = .29). Perceived relaxation during meditation and during yoga, in turn, correlated highly with each other (r = .55), as did perceived wakefulness during meditation and during yoga (r = .58). Relaxation during yoga also correlated moderately with wakefulness during yoga (r = .37), and coherence of yoga movements with the breath (r = .36). Perceived ease of ethical exercises had smaller correlations with relaxation during meditation (r = .24) and yoga (r = .26). Thus, there seems to have been a relevant coherence between different subjective experiences and different components of practice. Our data do not allow for causal inferences. However, we can conclude that on "good" days participants perceived all of their home exercises as easier and were more relaxed and awake during meditation and/or yoga. Surprisingly, meditation or yoga practice duration did not correlate with any of the subjective experience variables. They did correlate with each other (r = .48), though, indicating that on days where participants practiced longer meditation, they also practiced longer yoga, and vice versa.

Section A6

Effects of Time of Meditation Practice

First, we split reported times of meditation practice into large bands, that is, morning (5–11 a.m.), midday (11 a.m.–3 p.m.), afternoon (3-6 p.m.), and evening (6p.m.–midnight). The majority of participants (74 %) had a preferred time band for meditation. The most popular time for meditating was during the evening (38 %), followed by morning (21 %), midday (12 %), and afternoon (2 %). There were no apparent differences in conditions regarding preference of meditation time. However, all participants also reported practicing regularly in more than one time band. Therefore, we included three dummy variables coding the four time bands into our multilevel model reported above. The evening band served as the reference category.

The most predictions stayed the same in the extended models (see Supplementary Material B—Tables B12 and B13). However, participants reported higher levels of well-being on days when they had meditated in the morning, b = 0.11, SE = 0.05, p = .031 The time of meditation did not influence perceived stress. However, individually higher meditation practice duration was now positively related to reduced stress (p < .05) and negatively to increased well-being (p < .01). This could imply that participants meditated less on days when they were feeling well, as they were consistently reporting during our weekly meetings, and more when they were feeling less stressed. Whether they felt less stressed *because* they meditated longer, we cannot tell from our data. However, many participants reported during our meetings that they used meditation to calm themselves down on stressful days, and in these cases, they needed to meditate longer than usual.
