# Supplementary material for: Differential Effects of Ethical Education, Physical Hatha Yoga, and Mantra Meditation on Well-Being and Stress in Healthy Participants—An Experimental Single-Case Study
Source: Front Psychol. 2021 Aug 5;12:672301. doi: 10.3389/fpsyg.2021.672301 (PMC8375679; doi:10.3389/fpsyg.2021.672301)
Supplement: Supplementary file 2 [file Data_Sheet_2.PDF]

Supplementary Material B

Additional Tables

Differential Effects of Ethical Education, Physical Hatha Yoga, and Mantra Meditation on Well-Being and Stress in Healthy Participants: An Experimental Single-Case Study

Table B1

Full Multi-Level Estimation Procedure for Well-Being

	Fixed effects					
	Model 0	Model 1	Model 1b	Model 2	Modell 3	Modell 4
	b (*SE*)	β (*SE*)	β (*SE*)	β (*SE*)	β (*SE*)	β (*SE*)
**Intercept**	4.14 (0.09)	0.0	0.0	0.0	0.0	0.0
**Level 1**						
Time		0.09*** (0.02)	0.08** (0.03)	0.08** (0.03)	0.08** (0.03)	0.08** (0.03)
**Level 2**						
Ethical education				0.01 (0.09)	0.07 (0.10)	-0.01 (0.12)
Physical yoga				0.01 (0.09)	0.04 (0.10)	-0.07 (0.12)
Practice time						0.15 (0.12)
Age						0.07 (0.12)
Gender						0.08 (0.10)
Occupation						-0.17 (0.13)
Baseline Length						0.00 (0.10)
**Interactions**						
Time * Ethics					0.07* (0.03)	0.07* (0.03)
Time * Yoga					0.03 (0.03)	0.03 (0.03)
	**Random effects**					
	Model 0	Modell 1	Model 1b	Model 2	Model 3	Model 4
**Level 1**						
Residual (σ^2_e)	0.58	0.61	0.57	0.57	0.57	0.57
Slope (σ^2_{u1})			0.04	0.04	0.04	0.04
**Level 2**						
Intercept (σ^2_{u0})	0.34	0.37	0.36	0.37	0.37	0.38
Deviance	5922.9	6091.3	6002.0	6002.1	5997.0	5993.2
Marginal R^2	0.01	0.01	0.01	0.01	.02	.05
Conditional R^2	0.38	0.38	0.41	0.42	.43	.45
ICC	0.38					
N	42	42	42	42	42	42
Observations	2538	2538	2538	2538	2538	2538

Note. Model 0 was the intercept-only model; Model 1 included time; Model 1b modelled random slopes for time; Model 2 included components, Model 3 modelled cross-level interactions; Model 4 included age, gender, and baseline length. *b* = unstandardized regression coefficient; β = standardized regression coefficient; *SE* = standard error.

* $p < .05$; ** $p < .01$; *** $p < .001$.

Table B2

Full Multi-Level Estimation Procedure for Stress

	Fixed effects					
	Model 0	Model 1	Model 1b	Model 2	Modell 3	Modell 4
	b (*SE*)	β (*SE*)	β (*SE*)	β (*SE*)	β (*SE*)	β (*SE*)
**Intercept**	2.52 (0.08)	0.0	0.0	0.0	0.0	0.0
**Level 1**						
Time		-0.06* (0.04)	-0.06 (0.05)	-0.06 (0.05)	-0.06 (0.05)	-0.06 (0.05)
**Level 2**						
Ethical education				-0.15 (0.12)	-0.15 (0.12)	0.02 (0.14)
Physical yoga				0.07 (0.12)	0.07 (0.12)	0.30* (0.14)
Practice time						-0.37* (0.15)
Age						-0.19 (0.15)
Gender						-0.09 (0.12)
Occupation						0.29 (0.16)
Baseline Length						0.07 (0.12)
**Interactions**						
Time * Ethics					0.01 (0.05)	0.01 (0.05)
Time * Yoga					-0.04 (0.05)	-0.04 (0.05)
	**Random effects**					
	Model 0	Modell 1	Model 1b	Model 2	Model 3	Model 4
**Level 1**						
Residual (σ^2_e)	0.20	0.44	0.40	0.40	0.40	0.40
Slope (σ^2_{u1})			0.04	0.04	0.04	0.04
**Level 2**						
Intercept (σ^2_{u0})	0.26	0.56	0.56	0.56	0.56	0.50
Deviance	569.6	860.7	852.2	850.4	849.7	839.8
Marginal R^2	0.00	0.00	0.00	0.03	.03	.15
Conditional R^2	0.56	0.56	0.60	0.61	.61	.64
ICC	0.56					
N	42	42	42	42	42	42
Observations	377	377	377	377	377	377

Note. Model 0 was the intercept-only model; Model 1 included time; Model 1b modelled random slopes for time; Model 2 included components, Model 3 modelled cross-level interactions; Model 4 included age, gender, and baseline length. *b* = unstandardized regression coefficient; β = standardized regression coefficient; *SE* = standard error.

* p < .05; ** p < .01; *** p < .001.

Table B3

*Mean Well-Being Scores With Standard Deviations for Baseline (A) and Treatment (B) Phases, and Tau-*U *Estimates With Respective Significance Levels and Type of Tau-*U *for Each Participant*

Case	*Mean* A	*Mean* B	*SD* A	*SD* B	Tau-*U*	Tau-*U* Significance	Tau-*U* Type
1	4.68	5.02	0.90	0.83	**0.246**	p = .190	A vs. B
2	3.54	3.85	0.81	1.13	-0.112	p = .181	A vs. B - Trend A + Trend B
3	3.71	2.91	0.65	0.57	**-0.458**	p = .000	A vs. B + Trend B
4	5.63	4.32	0.39	0.78	**-0.511**	p = .000	A vs. B + Trend B
5	4.16	4.46	0.65	0.69	**0.237**	p = .220	A vs. B
6	3.87	3.85	0.40	0.62	0.125	p = .120	A vs. B + Trend B
7	3.80	3.58	0.47	0.61	-0.163	p = .068	A vs. B - Trend A + Trend B
8	2.90	3.19	0.73	0.89	0.158	p = .523	A vs. B
9	3.58	3.69	1.31	1.04	0.063	p = .687	A vs. B
10	4.83	4.90	0.77	0.75	0.070	p = .686	A vs. B
11	3.20	3.18	0.70	0.54	**0.212**	p = .011	A vs. B - Trend A + Trend B
12	3.14	3.89	0.74	0.49	**0.359**	p = .000	A vs. B + Trend B
13	3.34	3.56	0.92	0.72	0.143	p = .442	A vs. B
14	4.53	5.17	0.50	0.81	**0.450**	p = .000	A vs. B + Trend B
15	5.16	5.62	0.32	0.32	**0.463**	p = .000	A vs. B + Trend B
16	4.08	4.65	0.77	0.73	**0.308**	p = .016	A vs. B - Trend A
17	3.90	3.78	0.43	0.41	-0.154	p = .301	A vs. B
18	4.10	4.81	0.97	0.94	**0.362**	p = .000	A vs. B - Trend A + Trend B
19	4.28	4.10	0.59	1.16	0.061	p = .699	A vs. B + Trend B
20	4.24	4.51	0.82	0.60	0.143	p =.541	A vs. B
21	3.48	3.90	0.68	0.85	**0.298**	p = .139	A vs. B
22	3.21	3.76	0.49	0.53	**0.426**	p = .000	A vs. B + Trend B
23	4.34	4.42	1.20	0.77	-0.002	p =.992	A vs. B
24	4.15	4.04	0.55	0.45	-0.142	p = .370	A vs. B
25	4.20	4.37	0.63	0.36	0.154	p = .121	A vs. B + Trend B

26	3.38	3.90	0.68	0.91	**0.273**	p = .004	A vs. B + Trend B
27	4.47	5.08	0.89	0.53	**0.252**	p = .002	A vs. B + Trend B
28	3.40	3.65	0.77	0.70	**0.221**	p = .010	A vs. B + Trend B
29	3.48	3.24	1.00	0.54	-0.141	p = .589	A vs. B
30	4.75	4.59	0.85	0.69	-0.135	p = .434	A vs. B
31	4.77	4.81	0.80	0.59	0.091	p = .258	A vs. B + Trend B
32	3.36	3.98	1.19	0.85	**0.332**	p = .060	A vs. B
33	4.52	4.49	0.87	1.05	**0.189**	p = .047	A vs. B + Trend B
34	3.38	3.83	0.58	0.77	**0.338**	p = .098	A vs. B
35	4.03	4.17	0.39	0.77	**0.232**	p= .365	A vs. B
36	4.32	5.07	1.16	0.65	**0.373**	p = .014	A vs. B
37	3.96	4.39	0.84	0.66	**0.249**	p = .170	A vs. B
38	4.35	4.04	0.45	0.58	**-0.304**	p = .039	A vs. B
39	3.76	4.10	0.90	1.22	**0.236**	p = .177	A vs. B
40	5.10	5.06	0.40	0.38	-0.087	p = .617	A vs. B
41	3.86	4.20	1.06	0.82	0.186	p = .427	A vs. B
42	3.29	3.36	1.09	0.68	-0.042	p = .788	A vs. B

Note. Effect sizes significant on $\alpha < 0.05$ level or greater 0.20 were printed in bold type.

Table B4

Correlation Matrix of Tau-U Effect Size Estimates for Well-Being and Stress and the Predictors Component, Age, Gender, Baseline Length, Occupation, and Total Practice Time as Predictors

		1	2	3	4	5	6	7	8
1	Tau-U well-being								
2	Tau-U stress	-.41							
3	Ethical education (y/n)	.36	-.02						
4	Physical yoga (y/n)	.04	-.10	.00					
5	Age	-.27	.10	-.19	-.04				
6	Gender	.09	.19	.17	-.06	.05			
7	Baseline	.01	.01	.03	.03	-.20	.12		
8	Occupation	-.14	.06	-.02	-.27	.66	.00	-.12	
9	Total practice time	.02	-.14	.49	.43	-.02	.02	-.07	.00

Table B5

*Regression Model for Tau-*U *Well-Being Estimates as Dependent Variable and Condition, Total Practice Time, Age, Gender, Occupation, and Baseline Lenght as Predictors (*df = *35)*

Variable	*b*	β	*SE*	*t*	*p*
(Intercept)	0.23	0.00	0.22	1.05	.303
ME condition	0.29	0.56	0.11	2.67	.006
MY condition	0.16	0.30	0.11	1.55	.066
MYE condition	0.27	0.51	0.13	1.98	.028
Total practice time	0.00	-0.23	0.00	-1.15	.260
Age	-0.01	-0.19	0.01	-0.86	.396
Gender	0.02	0.04	0.10	0.25	.805
Occupation	0.00	0.00	0.15	-0.01	.989
Baseline length	0.00	-0.07	0.01	-0.47	.642

Table B6

Multilevel Regression Estimates for Well-Being Scores as Dependent Variable and Time, Conditions, Total Practice Time, Age, Gender, Occupation, and Baseline Length as Predictors

Variable	β	*SE*	*df*	*t*	*p*
Time	0.08	0.03	2492	2.64	.004
ME condition	0.06	0.14	33	0.44	.661
MY condition	0.01	0.13	33	0.05	.958
MYE condition	-0.08	0.16	33	-0.48	.634
Total practice time	0.16	0.13	33	1.26	.216
Age	0.07	0.13	33	0.57	.572
Gender	0.08	0.10	33	0.80	.432
Occupation	-0.19	0.14	33	-1.37	.180
Baseline Length	0.00	0.10	33	0.02	.987
Time * ME condition	0.13	0.04	2492	3.12	.001
Time * MY condition	0.09	0.04	2492	2.28	.011
Time * MYE condition	0.09	0.04	2492	2.30	.011

Table B7

*Mean Stress Scores With Standard Deviations for Baseline (A) and Treatment (B) Phases, and Tau-*U *Estimates With Respective Significance Levels and Type of Tau-*U *for Each Participant*

Case	***Mean*** **A**	***Mean*** **B**	***SD*** **A**	***SD*** **B**	**Tau-*U***	**Tau-*U* Significance**	**Tau-*U* Type**
1	1.75	1.86	0.64	0.67	0.067	p = .884	Tau-U A vs. B - Trend A
2	2.60	2.43	0.28	0.48	-0.118	p = .795	Tau-U A vs. B - Trend A
3	2.90	3.24	0.57	0.30	**0.294**	p = .511	Tau-U A vs. B - Trend A
4	2.30	2.66	n.d.	0.50	**0.267**	p = .444	Tau-U A vs. B + Trend B
5	2.20	2.67	0.00	0.47	**0.370**	p = .206	Tau-U A vs. B + Trend B
6	2.73	2.68	0.29	0.18	**-0.222**	p = .541	Tau-U A vs. B - Trend A + Trend B
7	2.90	2.95	n.d.	0.36	0.028	p = .914	Tau-U A vs. B - Trend A
8	3.50	3.55	n.d.	0.13	**0.300**	p = .448	Tau-U A vs. B + Trend B
9	4.07	3.34	0.35	0.53	**-0.527**	p = .023	Tau-U A vs. B - Trend A + Trend B
10	1.40	1.84	0.28	0.49	**0.588**	p = .191	Tau-U A vs. B - Trend A
11	2.50	2.39	n.d.	0.34	**0.378**	p = .125	Tau-U A vs. B + Trend B
12	2.40	2.29	n.d.	0.21	**-0.393**	p = .170	Tau-U A vs. B + Trend B
13	2.90	3.00	0.14	0.63	**0.200**	p = .534	Tau-U A vs. B + Trend B
14	2.40	1.91	n.d.	0.36	**-0.733**	p = .003	Tau-U A vs. B + Trend B
15	1.40	1.34	0.28	0.27	**-0.235**	p = .596	Tau-U A vs. B - Trend A
16	2.17	1.90	0.32	0.27	**-0.292**	p = .427	Tau-U A vs. B - Trend A
17	2.73	2.84	0.25	0.23	0.148	p = .684	Tau-U A vs. B - Trend A
18	1.73	1.91	0.55	0.54	-0.145	p = .529	Tau-U A vs. B - Trend A + Trend B
19	2.40	2.39	0.28	0.78	**0.267**	p = .281	Tau-U A vs. B - Trend A + Trend B
20	2.50	2.03	n.d.	0.23	**-0.714**	p = .012	Tau-U A vs. B + Trend B
21	3.10	2.63	0.00	0.32	**-0.333**	p = .435	Tau-U A vs. B + Trend B
22	3.50	2.84	0.14	0.32	**-0.743**	p = .006	Tau-U A vs. B + Trend B
23	2.50	2.03	n.d.	0.41	**-0.444**	p = .069	Tau-U A vs. B + Trend B
24	2.80	3.06	0.42	0.38	**0.294**	p = .513	Tau-U A vs. B - Trend A
25	3.03	3.02	0.06	0.26	**-0.214**	p = .451	Tau-U A vs. B - Trend A + Trend B

26	2.40	1.76	0.28	0.28	**-0.694**	p = .009	Tau-U A vs. B - Trend A + Trend B
27	2.65	2.51	0.07	0.55	**-0.389**	p = .098	Tau-U A vs. B + Trend B
28	3.00	2.34	n.d.	0.44	**-0.607**	p = .030	Tau-U A vs. B + Trend B
29	2.90	3.03	n.d.	0.36	**0.400**	p = .327	Tau-U A vs. B + Trend B
30	2.25	2.52	0.64	0.52	**0.345**	p = .133	Tau-U A vs. B - Trend A + Trend B
31	2.00	1.89	0.26	0.42	**-0.409**	p = .061	Tau-U A vs. B - Trend A + Trend B
32	3.30	3.13	0.57	0.45	**0.250**	p = .345	Tau-U A vs. B - Trend A + Trend B
33	3.20	3.01	n.d.	0.95	-0.107	p = .708	Tau-U A vs. B + Trend B
34	2.40	2.77	n.d.	0.06	**0.833**	p = .071	Tau-U A vs. B + Trend B
35	2.60	2.43	n.d.	0.39	**0.333**	p = .293	Tau-U A vs. B + Trend B
36	3.10	2.43	0.53	0.63	**-0.625**	p = .094	Tau-U A vs. B - Trend A
37	2.65	2.57	0.35	0.35	**-0.250**	p = .345	Tau-U A vs. B - Trend A + Trend B
38	1.63	2.73	0.21	0.41	**0.867**	p = .018	Tau-U A vs. B - Trend A
39	3.05	3.12	0.92	0.42	0.091	p = .849	Tau-U A vs. B - Trend A
40	1.55	1.45	0.07	0.15	**-0.412**	p = .346	Tau-U A vs. B - Trend A
41	2.60	1.80	n.d.	0.19	**-1.000**	p = .114	Tau-U A vs. B - Trend A
42	3.33	2.84	0.21	0.79	-0.167	p = .654	Tau-U A vs. B - Trend A

Note. Effect sizes significant on α < 0.05 level or greater 0.20 were printed in bold type. n.d. = not defined (because there was only one measurement in phase A).

Table B8

*Regression Model for Tau-*U *Stress Estimates as Dependent Variable and Condition, Total Practice Time, Age, Gender, Occupation, and Baseline Lenght as Predictors (*df *= 35)*

Variable	*b*	β	*SE*	*t*	*p*
(Intercept)	0.17	0.00	0.45	0.38	.708
ME condition	-0.25	-0.25	0.22	-1.10	.139
MY condition	-0.28	-0.27	0.21	-1.28	.105
MYE condition	0.06	0.06	0.27	0.22	.413
Total practice time	0.00	-0.22	0.00	-1.00	.326
Age	0.00	0.02	0.01	0.07	.945
Gender	0.24	0.20	0.20	1.24	.225
Occupation	0.12	0.10	0.30	0.42	.679
Baseline length	0.00	-0.01	0.01	-0.06	.952

Table B9

Multilevel Regression Estimates for Stress Scores as Dependent Variable and Time, Conditions, Total Practice Time, Age, Gender, Occupation, and Baseline Length as Predictors

Variable	β	*SE*	*df*	*t*	*p*
Time	-0.06	0.05	331	-1.25	.107
ME condition	-0.22	0.15	33	-1.45	.157
MY condition	0.04	0.14	33	0.31	.756
MYE condition	0.31	0.18	33	1.71	.097
Total practice time	-0.42	0.14	33	-2.93	.006
Age	-0.24	0.14	33	-1.67	.104
Gender	-0.06	0.11	33	-0.54	.596
Occupation	0.37	0.15	33	2.39	.023
Baseline Length	0.07	0.11	33	0.62	.541
Time * ME condition	-0.05	0.06	331	-0.95	.172
Time * MY condition	-0.09	0.06	331	-1.64	.051
Time * MYE condition	-0.03	0.06	331	-0.52	.303

Table B10

Multilevel Regression Estimates for Well-Being Scores as Dependent Variable and Time, Effective Component, Total Practice Time, Age, Gender, Occupation, Baseline Length, and Meditation Practice Variables as Predictors

Variable	*b*	*SE*	*df*	*t*	*p*
Time	-0.12	0.06	1472	-1.97	.024
Ethical education (y/n)	0.00	0.30	34	0.00	.997
Physical yoga (y/n)	-0.40	0.29	34	-1.37	.181
Total practice time	0.00	0.00	34	0.76	.453
Age	0.00	0.02	34	0.16	.874
Gender (male)	0.21	0.27	34	0.78	.440
Occupation (employed)	-0.48	0.40	34	-1.20	.237
Baseline length	0.01	0.02	34	0.34	.732
Meditation practice duration	0.00	0.00	1472	-1.27	.203
Meditation ease	0.06	0.02	1472	2.37	.018
Meditation wakefulness	0.22	0.02	1472	14.01	.000
Meditation relaxation	0.16	0.02	1472	7.42	.000
Time * Ethical education	0.04	0.07	1472	0.51	.303
Time * Physical yoga	0.12	0.07	1472	1.76	.039

Table B11

Multilevel Regression Estimates for Stress Scores as Dependent Variable and Time, Effective Component, Total Practice Time, Age, Gender, Occupation, Baseline Length, and Meditation Practice Variables as Predictors

Variable	*b*	*SE*	*df*	*t*	*p*
Time	-0.04	0.08	185	-0.52	.302
Ethical education (y/n)	-0.29	0.32	34	-0.90	.376
Physical yoga (y/n)	0.56	0.32	34	1.73	.092
Total practice time	0.48	0.31	34	1.52	.138
Age	-0.01	0.01	34	-0.95	.348
Gender (male)	-0.11	0.22	34	-0.50	.617
Occupation (employed)	0.48	0.31	34	1.52	.138
Baseline length	0.01	0.01	34	0.96	.344
Meditation practice duration	-0.01	0.01	185	-1.88	.061
Meditation ease	0.03	0.04	185	0.65	.516
Meditation wakefulness	-0.03	0.03	185	-1.10	.275
Meditation relaxation	-0.08	0.04	185	-2.06	.041
Time * Ethical education	0.09	0.09	185	1.03	.153
Time * Physical yoga	-0.08	0.09	185	-0.84	.200

Table B12

Multilevel Regression Estimates for Well-Being Scores as Dependent Variable and Time, Effective Component, Total Practice Time, Age, Gender, Occupation, Baseline Length, Meditation Practice and Meditation Time Variables as Predictors

Variable	*b*	*SE*	*df*	*t*	*p*
Time	-0.14	0.06	1420	-2.30	.011
Ethical education (y/n)	-0.05	0.30	34	-0.17	.866
Physical yoga (y/n)	-0.41	0.30	34	-1.39	.175
Total practice time	0.00	0.00	34	0.72	.476
Age	0.00	0.02	34	0.20	.842
Gender (male)	0.20	0.27	34	0.73	.469
Occupation (employed)	-0.52	0.40	34	-1.31	.198
Baseline length	0.01	0.02	34	0.33	.741
Meditation practice duration	-0.01	0.00	1420	-2.59	.010
Meditation ease	0.06	0.02	1420	2.71	.007
Meditation wakefulness	0.23	0.02	1420	13.96	.000
Meditation relaxation	0.16	0.02	1420	7.34	.000
Meditation time (5-11 am)	0.11	0.05	1420	2.16	.031
Meditation time (11-3 pm)	0.09	0.05	1420	1.77	.077
Meditation time (3-6 pm)	0.00	0.06	1420	-0.05	.958
Time * Ethical education	0.06	0.07	1420	0.85	.198
Time * Physical yoga	0.13	0.07	1420	1.79	.037

Table B13

Multilevel Regression Estimates for Stress Scores as Dependent Variable and Time, Effective Component, Total Practice Time, Age, Gender, Occupation, Baseline Length, Meditation Practice and Meditation Time Variables as Predictors

Variable	*b*	*SE*	*df*	*t*	*p*
Time	-0.04	0.08	177	-0.48	.314
Ethical education (y/n)	-0.31	0.32	34	-0.96	.344
Physical yoga (y/n)	0.58	0.32	34	1.78	.084
Total practice time	0.00	0.00	34	-2.07	.046
Age	-0.01	0.01	34	-0.92	.364
Gender (male)	-0.13	0.22	34	-0.59	.558
Occupation (employed)	0.49	0.32	34	1.53	.136
Baseline length	0.01	0.01	34	0.92	.364
Meditation practice duration	-0.02	0.01	177	-2.27	.024
Meditation ease	0.03	0.04	177	0.64	.521
Meditation wakefulness	-0.04	0.03	177	-1.27	.206
Meditation relaxation	-0.08	0.04	177	-2.18	.030
Meditation time (5-11 am)	0.09	0.09	177	0.96	.338
Meditation time (11-3 pm)	0.01	0.10	177	0.08	.937
Meditation time (3-6 pm)	0.01	0.10	177	0.08	.933
Time * Ethical education	0.11	0.09	177	1.17	.245
Time * Physical yoga	-0.09	0.09	177	-0.96	.339
