# Supplementary material for: Differential Effects of Ethical Education, Physical Hatha Yoga, and Mantra Meditation on Well-Being and Stress in Healthy Participants—An Experimental Single-Case Study
Source: Front Psychol. 2021 Aug 5;12:672301. doi: 10.3389/fpsyg.2021.672301 (PMC8375679; doi:10.3389/fpsyg.2021.672301)
Supplement: Supplementary file 3 [file Data_Sheet_3.PDF]

Supplementary Material C

Additional Figures

Differential Effects of Ethical Education, Physical Hatha Yoga, and Mantra Meditation on Well-Being and Stress in Healthy Participants: An Experimental Single-Case Study

Figure C1

Smoothed Loess Trend Lines of the Average Number of Minutes Spent Practicing Meditation Each Day in Four Conditions

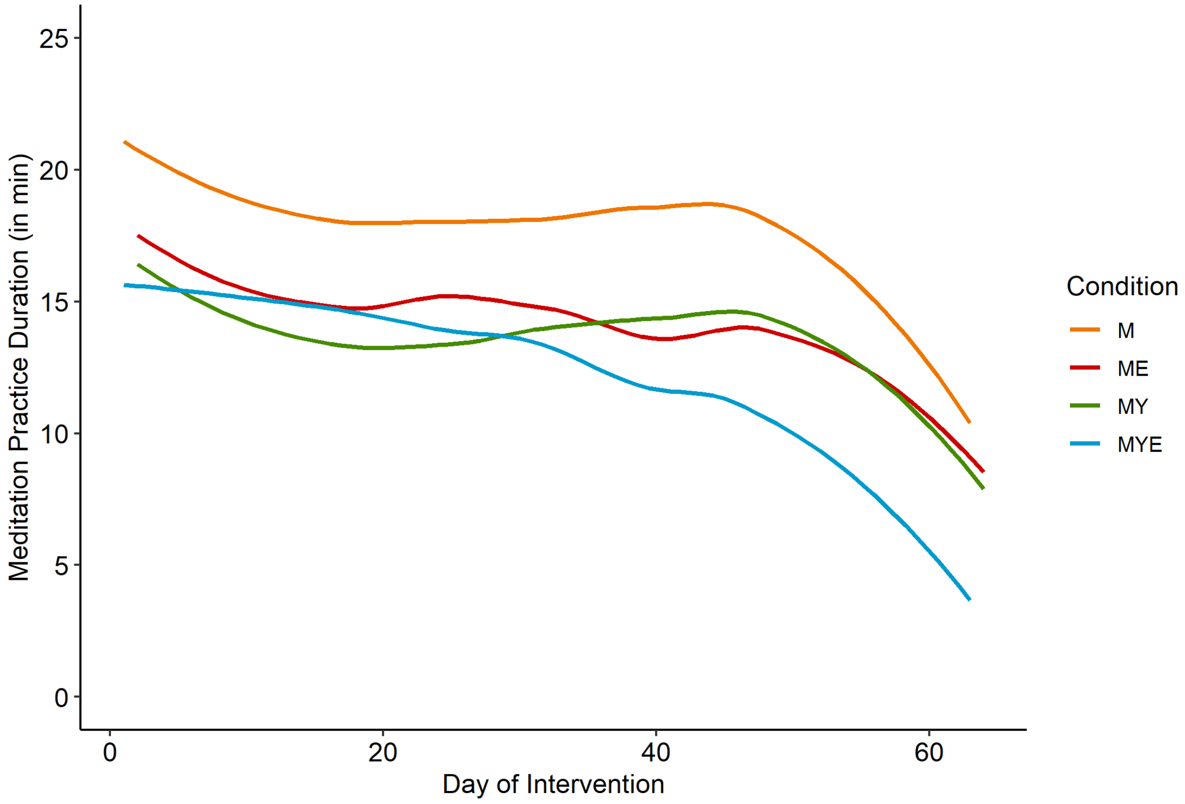

Figure C2

Smoothed Loess Trend Lines of the Average Number of Minutes Spent Practicing Yoga Exercises Each Day in Two Conditions

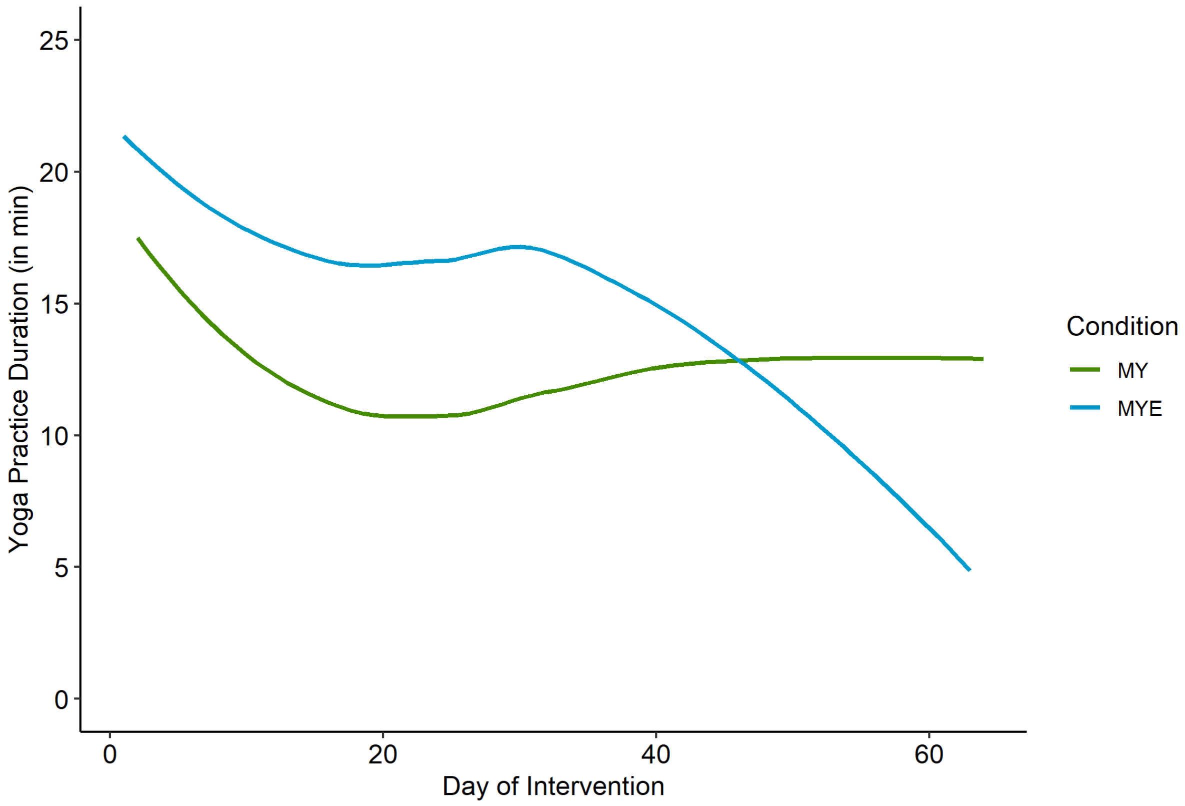

Figure C3

Smoothed Loess Trend Lines of the Proportion of Participants Engaging in Ethical Practice Each Day in Two Conditions

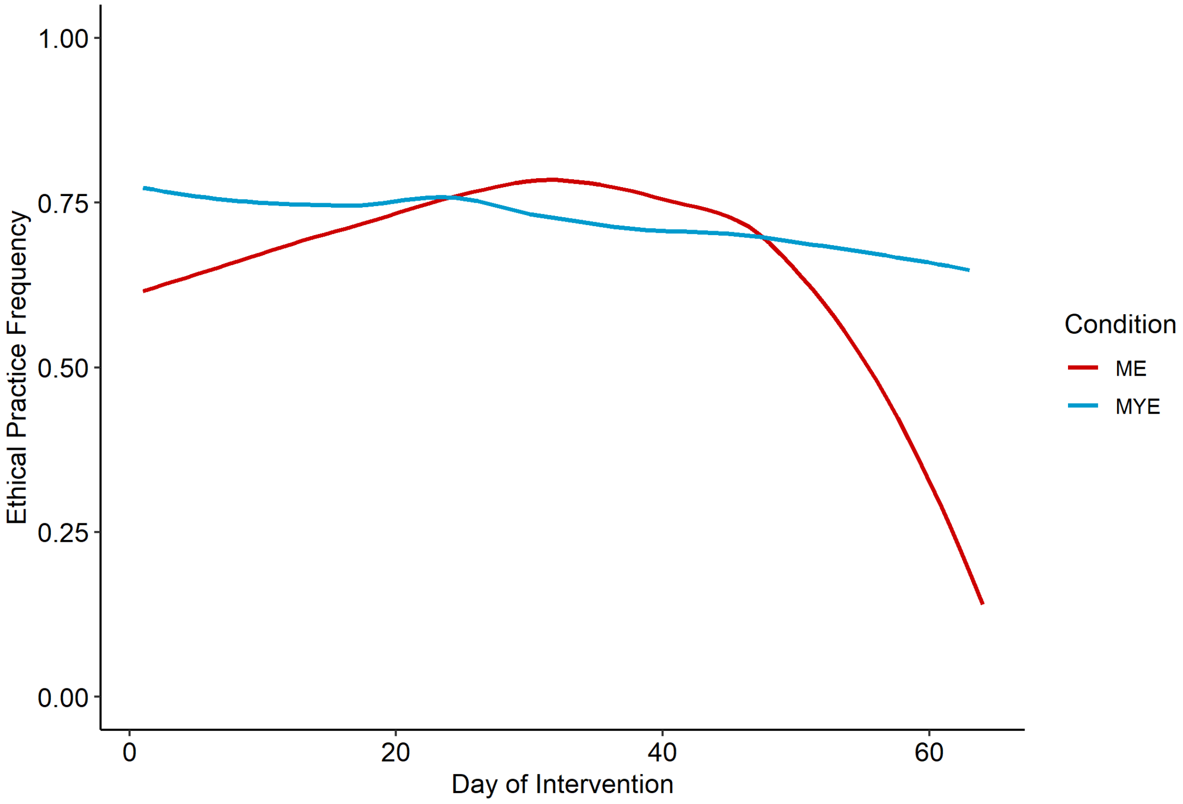

Figure C4

Ratings of Perceived Meditation Ease for Each Participant with Corresponding Regression Lines

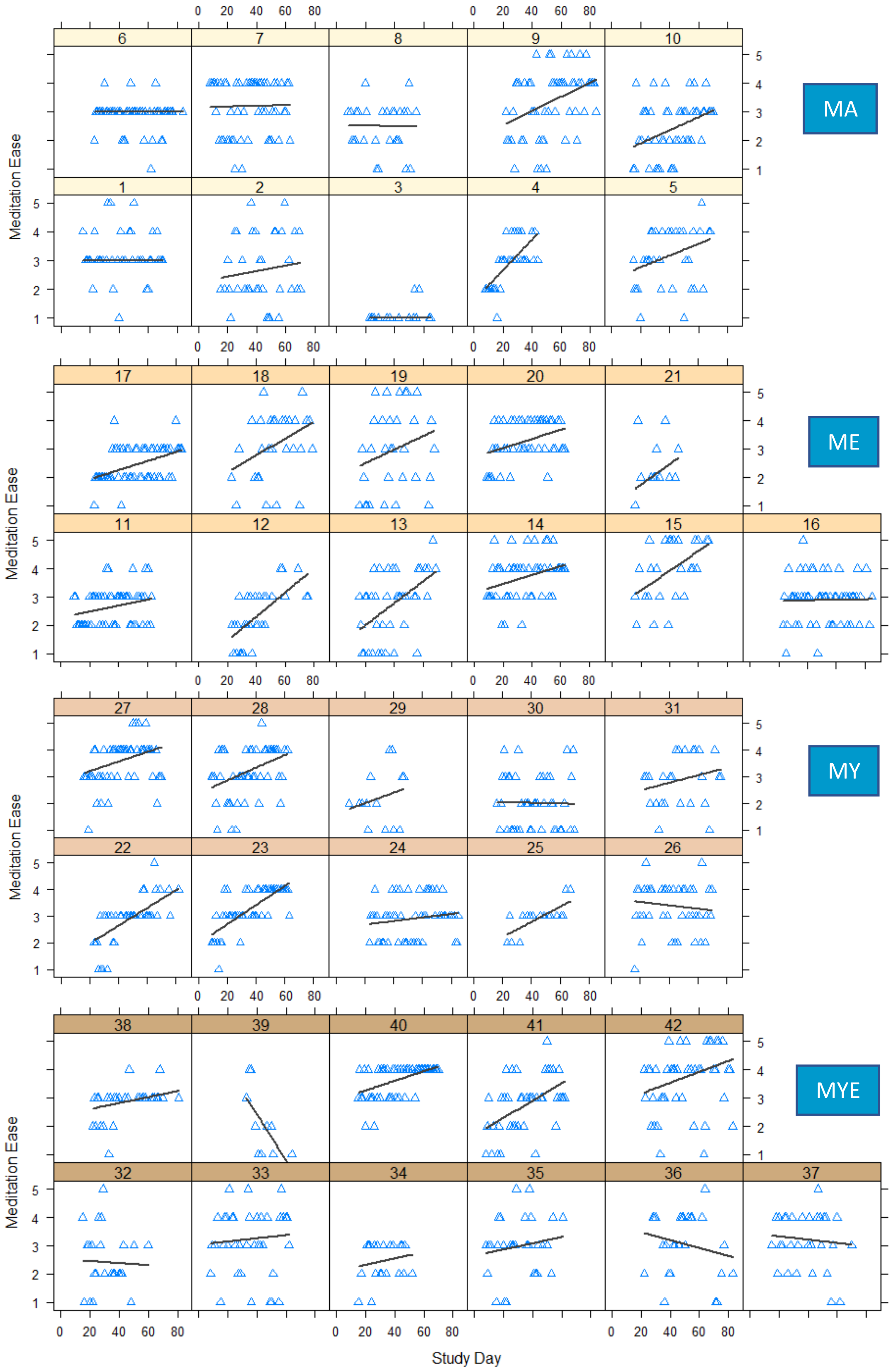

Note. MA = mantra meditation only, ME = meditation and ethical education, MY = meditation and physical yoga, MYE = meditation, physical yoga, and ethical education

Figure C5

Ratings of Relaxation During Meditation for Each Participant with Corresp. Regression Lines

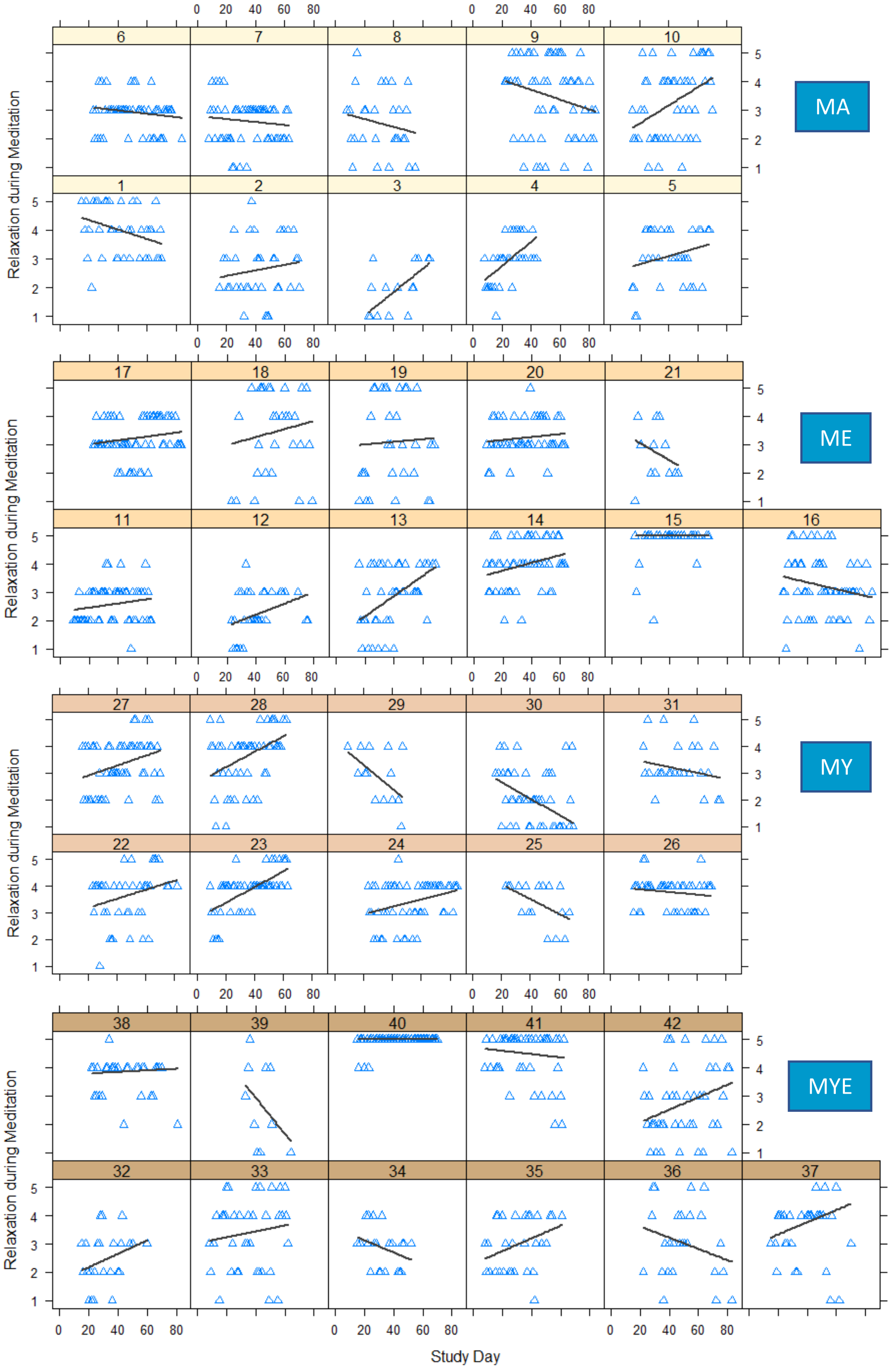

Note. MA = mantra meditation only, ME = meditation and ethical education, MY = meditation and physical yoga, MYE = meditation, physical yoga, and ethical education

Figure C6

Ratings of Wakefulness During Meditation for Each Participant with Corresp. Regression Lines

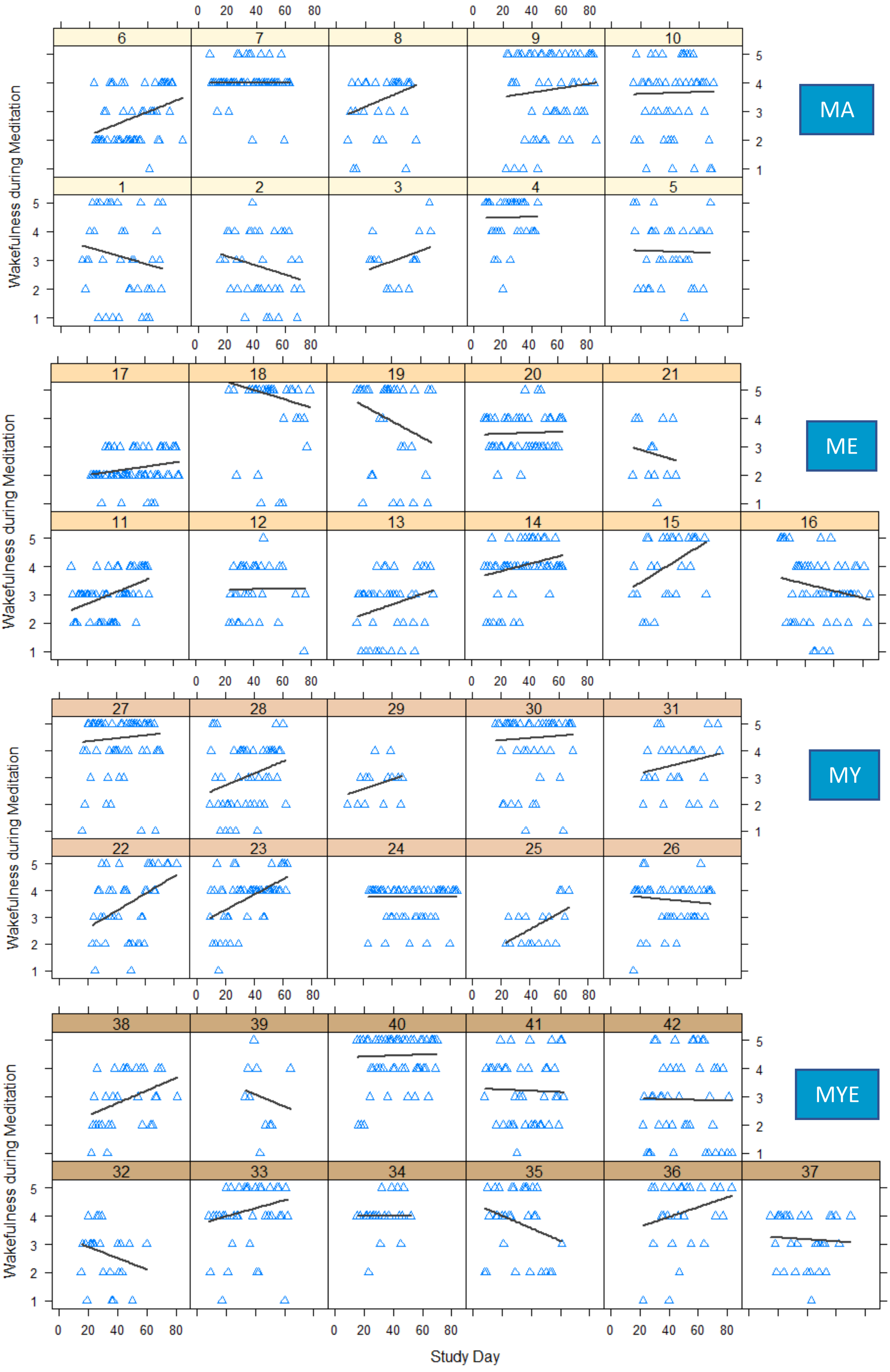

Note. MA = mantra meditation only, ME = meditation and ethical education, MY = meditation and physical yoga, MYE = meditation, physical yoga, and ethical education

Figure C7

Ratings of Perceived Yoga Ease for Each Participant with Corresponding Regression Lines

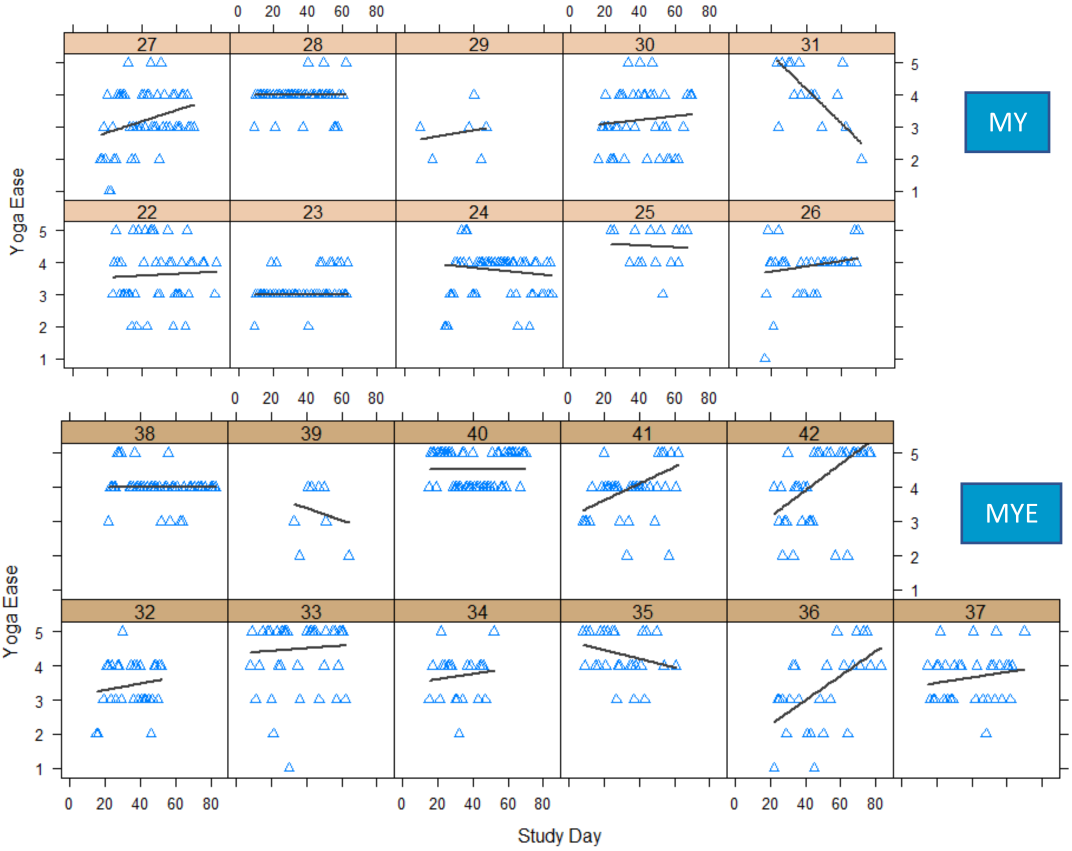

Note. MY = meditation and physical yoga, MYE = meditation, physical yoga, and ethical education

Figure C8

Ratings of Relaxation During Yoga for Each Participant with Corresponding Regression Lines

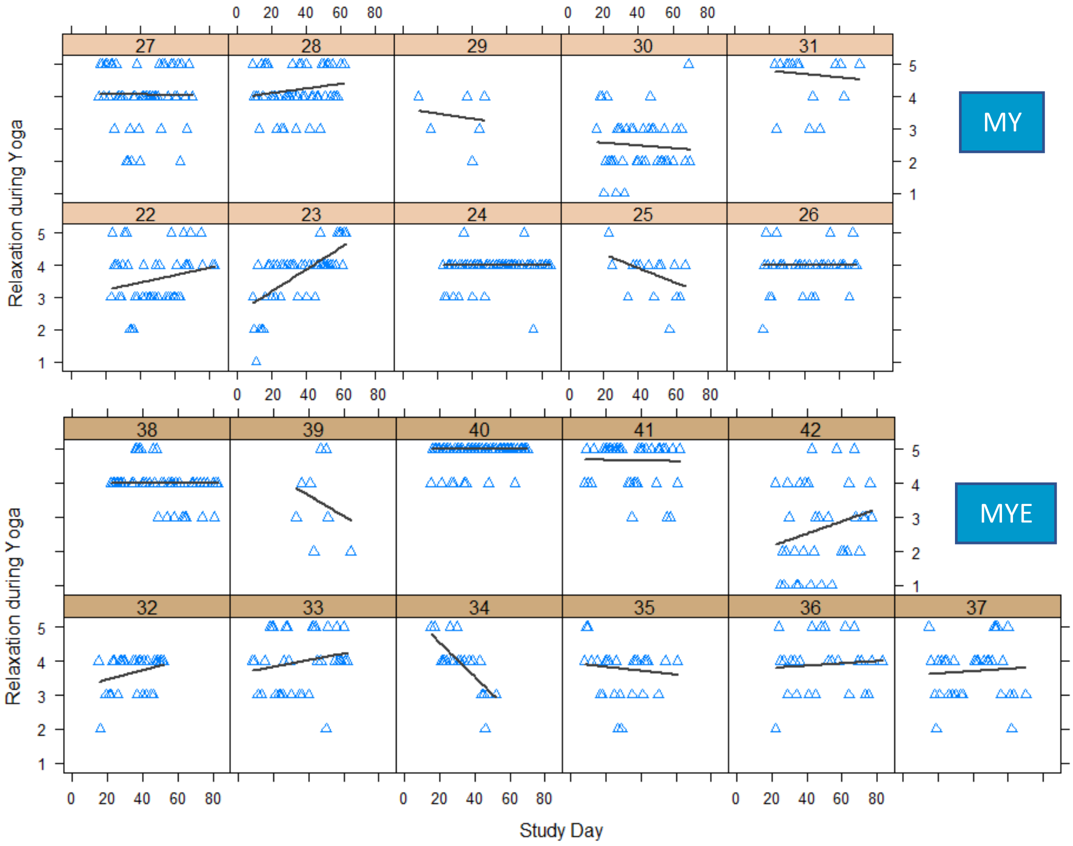

Note. MY = meditation and physical yoga, MYE = meditation, physical yoga, and ethical education

Figure C9

Ratings of Wakefulness During Yoga for Each Participant with Corresponding Regression Lines

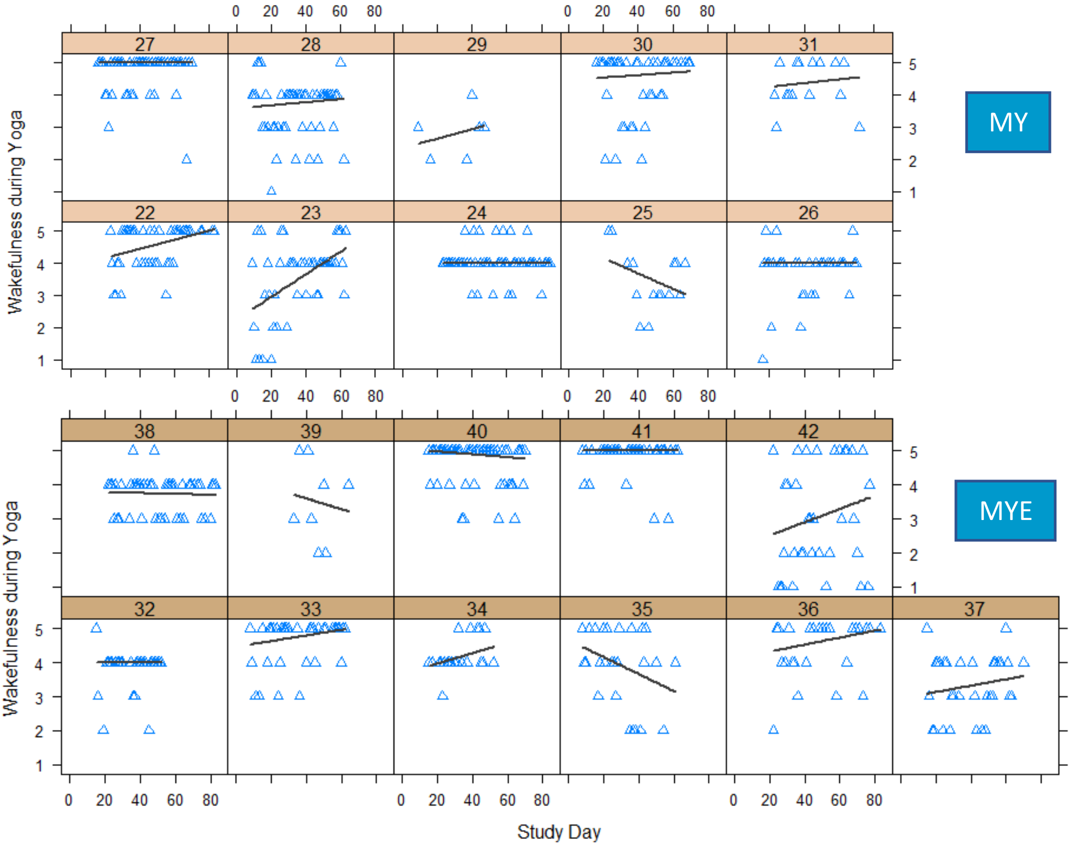

Note. MY = meditation and physical yoga, MYE = meditation, physical yoga, and ethical education

Figure C10

Ratings of Perceived Coherence of Yoga Exercises with Breathing for Each Participant with Corresponding Regression Lines

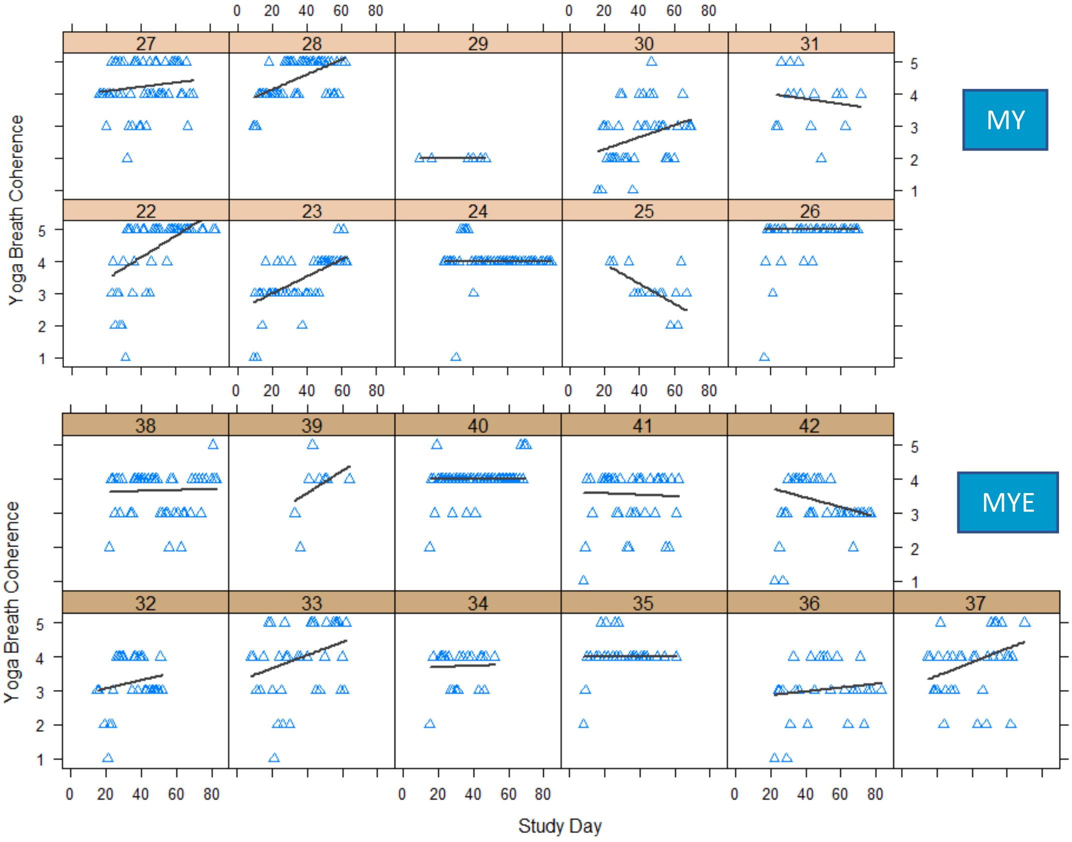

Note. MY = meditation and physical yoga, MYE = meditation, physical yoga, and ethical education

Figure C11

Ratings of Perceived Ease of Ethical Practice for Each Participant with Corresponding Regression Lines

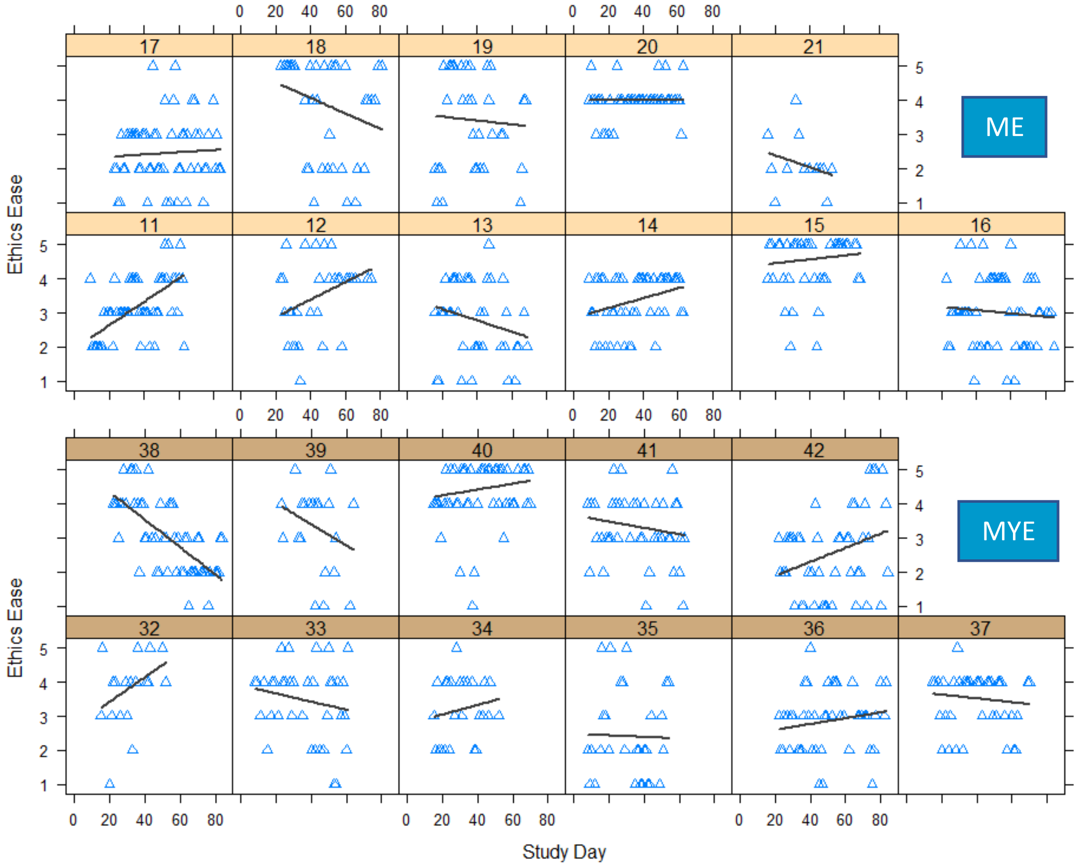

Note. ME = meditation and ethical education, MYE = meditation, physical yoga, and ethical education
